# Time-deterministic cryo-optical microscopy

**DOI:** 10.1038/s41377-025-01941-8

**Published:** 2025-08-23

**Authors:** Kosuke Tsuji, Masahito Yamanaka, Yasuaki Kumamoto, Shoko Tamura, Wakana Miyamura, Toshiki Kubo, Kenta Mizushima, Kakeru Kono, Hanae Hirano, Momoko Shiozaki, Xiaowei Zhao, Heqi Xi, Kazunori Sugiura, Shun-ichi Fukushima, Takumi Kunimoto, Yoshino Tanabe, Kentaro Nishida, Kentaro Mochizuki, Yoshinori Harada, Nicholas I. Smith, Rainer Heintzmann, Zhiheng Yu, Meng C. Wang, Takeharu Nagai, Hideo Tanaka, Katsumasa Fujita

**Affiliations:** 1 Department of Applied Physics, Graduate School of Engineering, The University of Osaka, Osaka, Japan; 2 Advanced Photonics and Biosensing Open Innovation Laboratory, AIST-Osaka University, Osaka, Japan; 3 Transdimensional Life Imaging Division, Institute for Open and Transdisciplinary Research Initiatives, The University of Osaka, Osaka, Japan; 4 Department of Pathology and Cell Regulation, Graduate School of Medical Science, Kyoto Prefectural University of Medicine, Kyoto, Japan; 5 Department of Dermatology, Course of Integrated Medicine, Graduate School of Medicine, The University of Osaka, Osaka, Japan; 6 Janelia Research Campus, Howard Hughes Medical Institute, Ashburn, VA USA; 7 Department of Biomolecular Science and Engineering, SANKEN, The University of Osaka, Osaka, Japan; 8 Immunology Frontier Research Center, The University of Osaka, Osaka, Japan; 9 Institute of Physical Chemistry and Abbe Center of Photonics, Friedrich-Schiller-University Jena, Jena, Germany; 10 Leibniz Institute of Photonic Technology, Jena, Germany

**Keywords:** Wide-field fluorescence microscopy, Ca2+ imaging, Super-resolution microscopy, Biophotonics, Imaging and sensing

## Abstract

Fluorescence microscopy enables the visualization of cellular morphology, molecular distribution, ion distribution, and their dynamic behaviors during biological processes. Enhancing the signal-to-noise ratio (SNR) in fluorescence imaging improves the quantification accuracy and spatial resolution; however, achieving high SNR at fast image acquisition rates, which is often required to observe cellular dynamics, still remains a challenge. In this study, we developed a technique to rapidly freeze biological cells in milliseconds during optical microscopy observation. Compared to chemical fixation, rapid freezing provides rapid immobilization of samples while more effectively preserving the morphology and conditions of cells. This technique combines the advantages of both live-cell and cryofixation microscopy, i.e., temporal dynamics and high SNR snapshots of selected moments, and is demonstrated by fluorescence and Raman microscopy with high spatial resolution and quantification under low temperature conditions. Furthermore, we also demonstrated that intracellular calcium dynamics can be frozen rapidly and visualized using fluorescent ion indicators, suggesting that ion distribution and conformation of the probe molecules can be fixed both spatially and temporally. These results confirmed that our technique can time-deterministically suspend and visualize cellular dynamics while preserving molecular and ionic states, indicating the potential to provide detailed insights into sample dynamics with improved spatial resolution and temporal accuracy in observations.

## Abstract (web summary)

Our cryo-optical microscopy rapidly freezes cells at an arbitrary timepoint during live imaging, enabling detailed observation of specific moments during dynamic events under cryogenic conditions.

## Graphical abstract

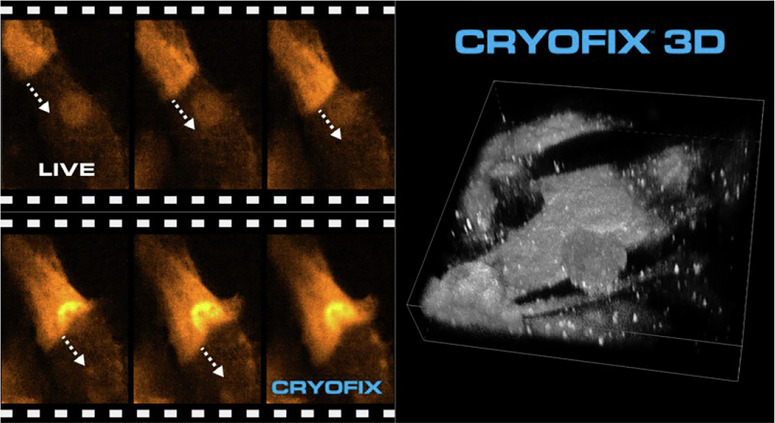

## Introduction

Fluorescence microscopy techniques are widely employed to study the cellular dynamics of biological events, as they enable visualizing molecular distributions, ion signaling, and molecular or protein interactions alongside cellular morphologies. Furthermore, the use of functional fluorescent probes^1–3^ and/or the incorporation of external stimulation techniques, such as electrical stimulation^4^, chemical condition changes^5^, and mechanical stimulation^6^, have further expanded the range of biological investigations possible with fluorescence microscopy.

Studying dynamic biological processes, where the distribution and state of molecules and ions changes over time, often requires fast image acquisition to accurately capture their co-localizations, behaviors, and interactions. While improving signal-to-noise ratio (SNR) offers distinct benefits, such as improved quantification accuracy and spatial resolution, achieving high SNR under short image acquisition times remains challenging. Additionally, the need for fast image acquisition also limits the applicability of various fluorescence imaging approaches, such as super-resolution imaging^7–12^, three-dimensional (3D) imaging, and fluorescence lifetime imaging, that would provide further detailed information but typically require relatively long image acquisition times, such as several hundred milliseconds to minutes.

In this study, we developed a technique utilizing a simple, easy-to-handle device to in situ rapidly freeze living cellular samples and their subcellular dynamics in millisecond order during observation using optical microscopy observations with high spatial and temporal resolutions. This technique visualizes the path of biological dynamics by tracing temporal information before fixation, allowing the rapid arrest of cellular morphologies, ion distributions, and chemical states at an arbitrary time during a biological event, while also enabling the acquisition of high SNR snapshots of them with extended exposure times under cryogenic conditions. Additionally, we also developed a method to control the timing of cryofixation with a precision of ± 10 ms. By combining this precise cryofixation timing control with an optical stimulation technique, we accurately determined the time interval between the initiation of biological events and cryofixation during optical microscopy observation. While rapid freezing-based techniques, such as pouring liquid cryogen over in vivo tissue or organ samples and subsequently performing freeze substitution, have previously been proposed and utilized for sample observation and analysis using optical/electron microscopy observations^13,14^, our technique specifically adapts rapid freezing for near-instantaneous immobilization of cellular samples and dynamics under optical microscopy observations, enabling subsequent cryogenic observations using various optical microscopy modalities.

Cryofixation has long been utilized to immobilize samples in the conditions close to their native states^15^, and has been frequently employed in the field of life science studies involving electron microscopy^16,17^. While the degree of immobilization required for optical microscopy is less stringent than for electron microscopy due to its lower spatial resolution, cryofixation still offers the distinct advantage over chemical fixation in preserving cellular structures^15,18,19^. This preservation is essential for analysis of cellular details using super-resolution fluorescence imaging techniques^20–27^. Moreover, cryofixation may preserve the chemical state and environment in samples, such as the redox state of molecules, pH, and ion concentration, which cannot be realized by traditional chemical fixation techniques, such as using paraformaldehyde^28–30^. The benefits of cryofixation in optical microscopy observations, including enhanced photostability of sample molecules and improved quantum yield, have also been recognized^31–36^. However, the optical properties of some fluorescent probes, such as photoswitching behaviors or the fluorescence intensity ratio within the fluorescence spectrum, may differ under cryogenic conditions compared to room temperature conditions or may show temperature dependencies^21,31,37^. Understanding these differences is crucial prior to performing cryogenic observations of samples using fluorescent probes^31,38,39^.

Recently, rapid freezing techniques of a sample on a microscope stage have been proposed and demonstrated, enabling cryofixation at specific time points during optical microscopy observations^40–43^. These techniques have successfully demonstrated the cryofixation of the movement of *Caenorhabditis elegans* during fluorescence imaging^41^, achieving cryofixation within 30 ms, as well as the cryofixation of mammalian cells during fluorescence imaging at time intervals from a few seconds to several minutes^43^. Subsequent cryogenic optical observations, including super-resolution or fluorescence lifetime imaging^40,43^, further demonstrated their potential. Advancing such rapid freezing techniques to enable the rapid immobilization of biological dynamics, especially at subcellular level, during optical microscopy observations with high spatial and temporal resolution, as studied in this research, significantly benefit a wide range of biological studies. High SNR and spatial-resolution snapshots of biological events at accurate moments, along with the recording of biological dynamic behaviors leading up to the immediate moment of cryofixation, are expected to provide deeper and more accurate insights into cellular functions, molecular distributions, molecular co-localization, and other aspects related to the dynamic biological events.

## Results

### On-stage chamber for rapid freezing

**Fig. 1:**
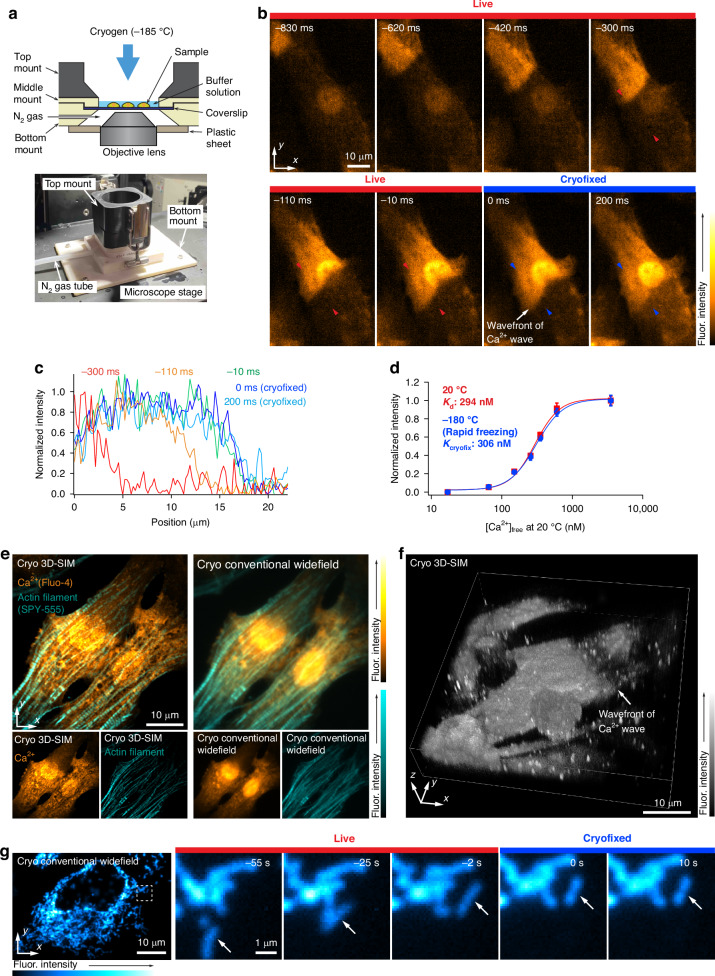
**On-stage freezing chamber, Cryofixation of cellular dynamics under microscopic observation, and cryogenic super-resolution imaging**. **a** Cross-sectional schematics and photograph of on-stage freezing chamber. **b** Cryofixation of Ca^2+^ wave propagating in the neonatal rat cardiomyocytes (frame rate: 100 frames s^-1^, Video S1). Ca^2+^ wave motion stopped at the frame of 0 ms by cryofixation. The white arrow in the image indicated the region where Ca^2+^ wave motion stopped. Post-cryofixation, the image contrast was predominantly preserved, with only slight discernible differences. Although further investigations are needed to elucidate the details of the slight difference in the image contrast before and after cryofixation, this may be attributed to variations in optical property of Fluo-4 in the cytoplasm and cellular organelles, contamination of cellular autofluorescence, which is also enhanced under cryogenic conditions, and the difference of physiochemical properties between Fluo-4 and cellular molecules under cryogenic conditions. If contamination of cellular autofluorescence is the case, employing spectral unmixing techniques or a calcium indicator with longer excitation and emission wavelengths would mitigate this issue significantly. Note that, as described in the Materials and Methods, the fluorescence intensity of each image was normalized for visualization purpose based on the fluorescence intensity of the selected area in the observed cells. **c** Fluorescence intensity profiles of the line indicated with the red and blue arrows in (**b**). These profiles show that the leading edge of the Ca^2+^ wave moves from left to right in this plot and is then arrested by cryofixation. These intensity line profiles were normalized to allow for comparison of the time variation in graph shapes at each line location. **d** Ca^2+^ titration curve of Fluo-4 under 20 °C and −180 °C (rapid freezing). This data was measured using Fluo-4 in a Ca^2+^ calibration buffer (“Materials and Methods”). After freezing the dissociation of calcium indicator is unlikely to occur in freezing conditions, as it does in liquid environments, we referred to the values calculated from the fitting function of the post-freezing data as *K*_cryofix_, instead of *K*_d_ to avoid confusion. It was confirmed that a *K*_d_ value similar to that in ref. ^45^ was obtained at the room temperature, and the shape of the Ca^2+^ titration curve was preserved after cryofixation. This result supports the preservation of the image contrast of the Fluo-4 loaded neonatal rat cardiomyocytes after cryofixation, as demonstrated in (**b**). **e** Cryogenic dual-color super-resolution and conventional widefield fluorescence images of Ca^2+^ distribution and actin filaments in the neonatal rat cardiomyocytes. **f** Cryogenic 3D super-resolution fluorescence image of Ca^2+^ distribution in the neonatal rat cardiomyocyte (Video S2). 3D visualization was produced by using alpha rendering (Nikon, NIS-Elements). **g** Cryogenic conventional widefield fluorescence images of a HeLa cell expressing DsRed in mitochondria (frame rate: 1 frames s^−1^, Video S3). The mitochondria moved at 37 °C and were immobilized by cryofixation. Although the cell shape was slightly deformed by cryofixation, it was not significant as shown in the enlarged views in this figure

We developed a sample-freezing chamber that can be placed on an inverted optical microscope, as shown in Fig. 1a (Materials and Methods). A liquid cryogen composed of propane and isopentane was introduced into the sample in the chamber so that the living cells were frozen by heat exchange between the cryogen and sample (Materials and Methods). The volume of buffer solution surrounding the cells was reduced specifically to facilitate rapid freezing, with no noticeable impact on biological events observed. With this method, the residual buffer height was estimated to be 6.7 ± 2.5 µm (Fig. S1). The cryogen was maintained on the sample to ensure that the sample remained at cryogenic temperature during optical observations. Our on-stage freezing chamber features an open-top design, enabling cell stimulation by altering buffer solution conditions prior to freezing. We confirmed that the drift velocity is only 31 nm s^−1^ after 30 s following rapid freezing, which is sufficiently low to avoid motion artifacts in fluorescence imaging using this on-stage freezing chamber (Fig. S2). We also confirmed that our on-stage freezing chamber maintained the sample temperature below the glass transition temperature of pure water (−136 °C)^44^ for about 3.3 min (Fig. S3), which is long enough for many applications using fluorescence imaging. To assess the extent of ice crystal formation with our freezing method, we performed cryo-transmission electronic microscopy (cryo-TEM) observation of HeLa cells, which were frozen by dropping the cryogen onto the sample. The cryo-TEM results confirmed that almost no ice crystal formation was observed within HeLa cells (Fig. S4). Given the spatial resolution of optical microscopy is on the order of several hundreds nm, such small ice crystal formation is unlikely to impact image quality or biological interpretation by optical microscopy. Supporting this, structured illumination microscopy (SIM)^12^ revealed no noticeable damage or notable morphological alternation in cells subjected to rapid freezing with our on-stage freezing chamber (Fig. S5).

### Cryofixation of cellular dynamics

Cryofixation was performed using a freezing chamber on a microscope stage to observe calcium activity, which is one of the most rapid cellular reactions. Figure 1b, c show a series of fluorescence images of neonatal rat cardiomyocytes loaded with a calcium-ion (Ca^2+^) indicator, Fluo-4, and fluorescence intensity profiles indicating the wavefront of the Ca^2+^ wave propagating in neonatal rat cardiomyocytes (Materials and Methods, Fig. S6 for another demonstration of cryofixation halting Ca^2+^ wave propagation rapidly). During the observation of the Ca^2+^ wave, the cryogen was introduced, and the wave motion was stopped (Video S1). This result shows that rapid freezing can preserve not only the cell morphology and Ca^2+^ distribution, but also the chemical state of the fluorescent indicator because the contrast of the image remains largely unaltered before and after fixation.

We confirmed the ability to perform Ca^2+^ observations at cryogenic temperatures, by measuring the fluorescence intensity over a range of Ca^2+^ concentrations, and found a sigmoidal response, similar in shape to that of room temperature, as shown in Fig. 1d. To compare the characteristics with those at room temperature, we then introduce an effective binding constant *K*_cryofix_, which refers to the inflection point in the response curve. On comparison with *K*_d_, we found that *K*_cryofix_ was almost unaltered by rapid freezing (294 vs 306 nM, Fig. 1d, Materials and Methods). Interestingly, the dissociation constant *K*_d_ of Fluo-4 has been reported to be temperature-dependent^45^. This indicates that if the freezing process is fast enough, molecular responses and spatio-temporal conditions are halted without passing through a thermal equilibrium state, implying the pre-freezing state is preserved upon rapid freezing. In this study, we also confirmed that the fluorescence intensity of Ca^2+^ indicator, Fluo-4, increased under cryogenic conditions; however, the magnitude of increase was different between the cell nucleus and cytoplasm (Fig. S7). Therefore, it is important to evaluate these regions independently when analyzing the spatial intensity distributions to avoid misinterpretation (Fig. S8, more detailed discussion in the Discussion section). During this experiment, the same optical system was used at room and cryogenic temperatures because the excitation and fluorescence spectra of Fluo-4 were not significantly different under these temperature conditions (Fig. S9).

The immobilization of the sample on the microscope stage can be fully exploited by various imaging techniques. Figure 1e shows a super-resolution fluorescence image obtained by three-dimensional (3D) structured illumination microscopy (3D-SIM)^12^ of Fluo-4 and SPY-555, an actin probe for live cells, in neonatal rat cardiomyocytes, demonstrating the super-resolution imaging of ions and associated cell behaviors that cannot be performed using conventional chemical fixation techniques (Fig. S10, “Materials and Methods”). Our result shows that the sarcomere lengths were slightly shorter in the high Ca^2+^ concentration region compared to the low Ca^2+^ concentration region^46^ (Fig. S11). It is important to note that the dark spots seen in the Ca^2+^ images under cryogenic conditions were already present before freezing (Fig. S12), confirming that they were not caused by the cryofixation process. These characteristic fluorescence intensity distributions were considered due to the accumulation or deposition of Ca^2+^ indicator within the cells^47^. Additionally, we confirmed that the spatial resolution of SIM is essentially maintained after cryofixation under our experimental conditions (Fig. S13).

We further performed 3D super-resolution imaging of the Ca^2+^ distribution in neonatal rat cardiomyocytes using 3D-SIM (Fig. 1f, Video S2, “Materials and Methods”), after Ca^2+^ wave propagation in neonatal rat cardiomyocytes was halted by cryofixation during observation with a conventional widefield fluorescence microscope (Video S2, Materials and Methods). This result demonstrates the capability of our technique to capture detailed 3D spatial information about rapid dynamic cellular processes at a precise moment, which has been significantly difficult to visualize under living conditions.

In addition to Ca^2+^ dynamics, we also confirmed that the cryofixation technique effectively immobilized the motions of various subcellular structures, such as the mitochondria (Fig. 1g, Video S3, Materials and Methods) and lysosomes (Video S3, Materials and Methods). To evaluate the extent of morphological changes by cryofixation, we calculated the measurement errors in 2-point lengths of fluorescence images before and after cryofixation, using a B-spline based non-rigid registration algorithm^48^ (Fig. S14). The measurement errors in length for mitochondria (Fig. 1g, Video S3) and lysosomes samples (Video S3) are similar to that in expansion microscopy reported in ref. ^48^. We also confirmed that, even at 15 min after freezing, no noticeable morphological changes caused by ice crystal formation were observed at the spatial resolution of widefield fluorescence microscopy used in this study, although ice crystals may form as the temperature increases (Fig. S15). This suggested that, at this level of spatial resolution, observations with longer exposure times are feasible and can be used to obtain fluorescence images with improved SNR.

### Time-deterministic cryofixation

**Fig. 2:**
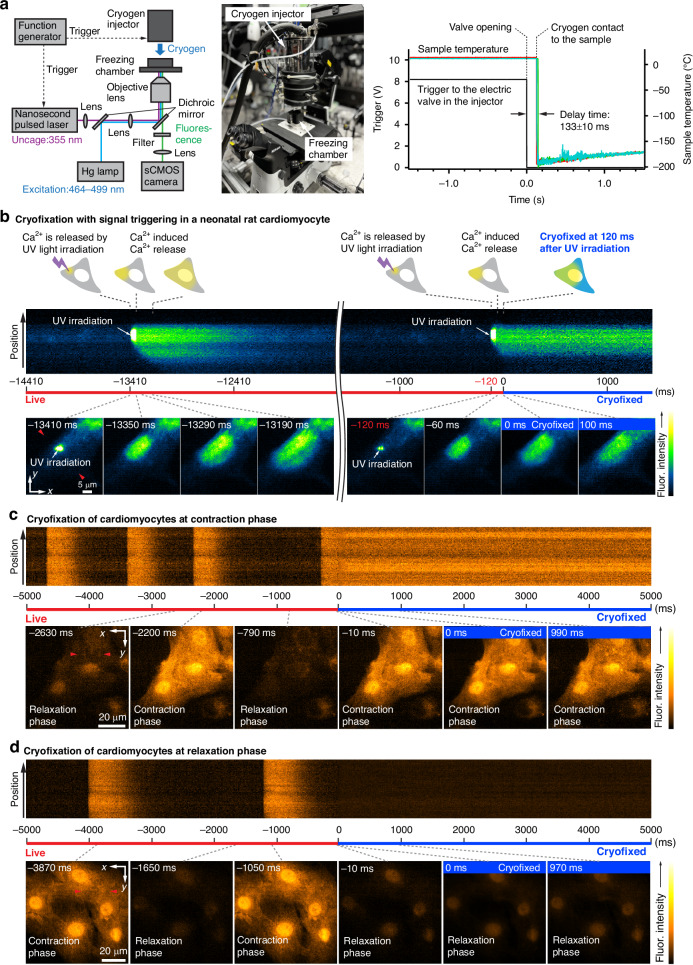
**Time-deterministic cryofixation of Ca**^**2+**^ **dynamics**. **a** Optical microscope setup equipped with a UV laser source for signal triggering and a precise cryogen injection control system (Materials and Methods). The timing of UV laser irradiation and cryogen injection was controlled by electrical trigger signals, which can be sent individually and synchronously or asynchronously to each device. The delay time between the trigger signal timing to cryogen injector and the cryogen injection timing onto samples was measured three times and plotted in red, green, and light blue, confirming the freezing time precision of ± 10 ms. 133 ms ± 10 ms delay was observed between triggering the cryogen injector and the moment when the cryogen reaches samples. By advancing the timing of the trigger signal for the cryogen injector relative to that for the UV laser light source, our system enables rapid freezing at any desired timing with the time precision of ± 10 ms under optical stimulation. **b** Schematics of the time course of Ca^2+^ signal triggering and cryofixation, and the time course of the change of fluorescence intensity of Fluo-4 at the line indicated by the red arrows in the frame of the X-Y image at −13,410 ms, and X-Y images at different times. Ca^2+^ propagation stopped at 120 ms after UV light irradiation by cryofixation (Video S4). **c**, **d** Cryofixation of the neonatal rat cardiomyocytes loaded with Fluo-4 during contraction and relaxation phases (Video S5). Liquid cryogen was provided onto the sample manually for C and D. The fluorescence images were acquired at a frame rate of 100 frames s^−1^ in all data

As shown above, the rapid cryofixation on the microscope stage allows us to precisely determine the time of fixation during observation, which provides temporal information on biological events just before and at the moment of cryofixation. Time-deterministic features are especially useful when combined with a technique that triggers biological events. We used a caged Ca^2+^ compound that can modulate the Ca^2+^ concentration in cells by light irradiation to trigger Ca^2+^ waves in neonatal rat cardiomyocytes before cryofixation^49^ (“Materials and Methods”). For the determination of the time between signal triggering and cryofixation, we developed a cryogen injector (Fig. 2a, Fig. S16, “Materials and Methods”), equipped with an externally triggerable electric valve, and enabled the injection of liquid cryogen onto a sample with a timing precision of ±10 ms (Fig. 2a). By using the optical setup in Fig. 2a, Ca^2+^ propagation in the cell was frozen at 120 ms after applying the trigger signal to the UV laser light source (Fig. 2b and Video S4). By referring to the video recorded immediately before, it is possible to determine the timing of the cell dynamics under observation, which are being observed in detail with the cryo-microscope. This technique can be combined with various external stimuli, such as electrical stimulation, chemical condition changes, and mechanical stimulation^4–6^.

As further examples, Fig. 2c, d show that we can selectively apply the cryogen at contraction or relaxation phases in neonatal rat cardiomyocytes, allowing the possibility to arrest heartbeat motion triggered by the cells (Video S5, “Materials and Methods”). Neonatal rat cardiomyocytes were loaded with Fluo-4, and an increase in the free Ca^2+^ concentration in the cytoplasm during the contraction of the heartbeat motion, known as a Ca^2+^ transient, was observed. As shown in Fig. 2c, d, neonatal rat cardiomyocytes were frozen at the rising (contraction phase) and falling (relaxation phase) time points of fluorescence intensity.

### Improved quantitative measurement

Molecules under cryogenic conditions exhibit an enhanced chemical stability, which can be exploited for quantitative measurements. Quantifying dynamic samples has several challenges under ambient conditions but becomes significantly easier under cryogenic conditions. While previous studies have reported increased optical stability of fluorescent probes under cryogenic conditions^31–36^, the optical properties and quantitative imaging performance of Fluo-4 and a ratiometric fluorescent Ca^2+^ probe, yellow cameleon 3.60 (YC3.60)^50^, have not been previously explored.

**Fig. 3:**
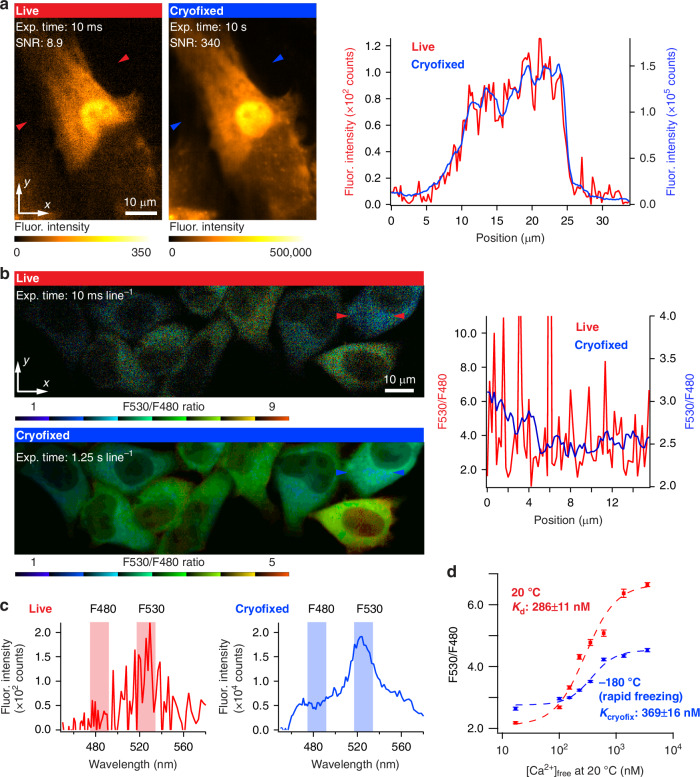
**Image quantification ability is improved by ultra-rapid fixation and cryogenic imaging**. **a** Fluorescence images of Fluo-4 loaded neonatal rat cardiomyocyte and fluorescence intensity line profiles of the lines indicated by the red and blue arrows in the fluorescence images. By increasing the exposure time by a factor of 1000, the SNR was improved from 8.9 to 340. The experimental data are the same as that shown in Fig. 1b. The fluorescence image of the cryofixed sample with the exposure equivalent to 10 s was generated by integrating 1000 fluorescence images with an exposure time of 10 ms under cryogenic conditions. **b** Ratiometric fluorescence images of HeLa cells expressing YC3.60 acquired with a slit-scanning hyperspectral fluorescence microscope before and after cryofixation. Here, we utilized MetaMorph software (Molecular Devices) to generate the ratiometric image using 8 shades of color in a look-up table shown at the bottom of the image. To facilitate the identification of cellular morphology, the intensities of each color in the image were adjusted to correspond with fluorescence intensities observed in the fluorescence image of Venus. Fluorescence ratio line profiles are also shown for the lines indicated by the red and blue arrows in the ratio images. The ratio values in the line profiles were calculated from the fluorescence intensity images of ECFP and Venus (Fig. S20). Similar to (**a**), the fluorescence image with the equivalent long exposure time under cryogenic conditions was generated by the integration of 125 fluorescence images with an exposure time of 10 ms line^−1^. **c** Representative fluorescence spectra of YC3.60 in the sample shown in (**b**). The increase of the exposure time allows us to improve SNR in the spectrum measurement. **d** Ca^2+^ titration curve of YC3.60 before and after cryofixation, which was measured by using YC3.60 dispersed in Ca^2+^ calibration buffer solution

As demonstrated in Fig. 3a, long-time exposure of a cryofixed sample produced a higher SNR in fluorescence imaging of Ca^2+^ distribution using Fluo-4, compared to limited exposure times in dynamics imaging under living conditions (Fig. 3a, “Materials and Methods”). Improved resistance of Fluo-4 to photobleaching under cryogenic conditions was also beneficial for improving the SNR in Ca^2+^ imaging (Fig. S17). The improvement in the SNR generates a corresponding increase in the quantification capability of the experiment. For example, a Ca^2+^ measurement accuracy of 0.2 nM can be achieved using Fluo-4 with 10 s exposure under −170 °C, demonstrating a 17-fold improvement compared to measurement conditions that could be used in live cell imaging (33 ms here, at 20 °C) (Fig. S18).

We also examined YC3.60, which is cyan fluorescent protein, ECFP, paired with yellow fluorescent protein, Venus, via Ca^2+^-sensitive calmodulin^50^. The optical property and imaging performance of YC3.60 under cryogenic conditions, like those of Fluo-4, have not been explored previously. Förster resonance energy transfer (FRET) between ECFP and Venus is facilitated by the capture of Ca^2+^ by calmodulin, and the ratio of the fluorescence intensity of ECFP and Venus can quantitatively measure the Ca^2+^ concentration in cells. Figure 3b shows a ratiometric fluorescence image of HeLa cells expressing YC3.60 in the cytosol and fluorescence ratio profiles obtained from the lines indicated by red and blue arrows in Fig. 3b, acquired using a slit-scanning hyperspectral fluorescence microscope (Fig. S19, “Materials and Methods”). The cells were cryofixed at 36 s after histamine stimulation. Long-time exposure under cryogenic conditions allowed us to measure the fluorescence spectrum of YC3.60 with a higher SNR, as shown in Fig. 3c, providing a high quantification capability for Ca^2+^ imaging. We also measured the calcium response of YC3.60 under the room temperature and cryogenic conditions at −180 °C after the rapid freezing mentioned above. We found that the YC3.60 performed effectively at cryogenic conditions over a useful range of calcium concentrations (several tens of nanomolars to micromolar range).

After demonstrating the applicability of the technique for calcium measurements, we compared the sigmoidal response curves of the probe after rapid freezing with that of room temperature and found a slight difference of the *K*_d_ and *K*_cryofix_ values, and a change in FRET efficiency (Fig. 3d, “Materials and Methods”). Although further investigations are needed to clarify the mechanism behind the change in fluorescence ratio after rapid freezing, this might be explained by changes in physicochemical properties, such as the increase in FRET efficiency due to the elongated fluorescence lifetime under low temperatures, a larger increase in the quantum yield of ECFP compared to that of Venus, changes in the emission spectrum shapes and possibly absorption spectrum shapes (Figs. S21–S23). Despite the changes in these physicochemical properties, ratiometric imaging at a high SNR for the quantification of Ca^2+^ concentration was achieved, indicating that various FRET-based probes can also be used with time-deterministic cryo-optical microscopy. The fluorescence ratio in cryogenic cell imaging was lower than those shown in the Ca^2+^ titration curve, which we attribute to the difference in the surrounding environmental conditions of YC3.60 in HeLa cells and the Ca^2+^ calibration buffer solution.

### Multimodal imaging capability with a different temporal resolution

**Fig. 4:**
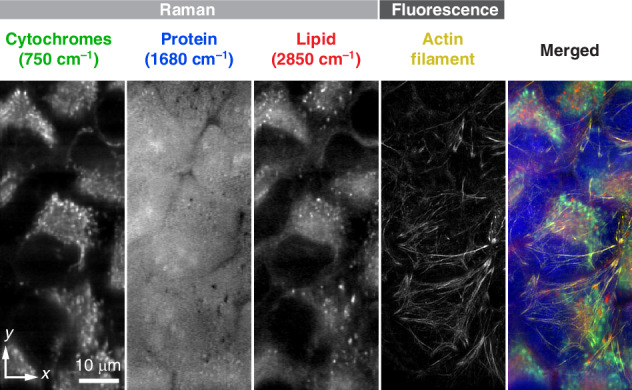
**Cryogenic spontaneous Raman and super-resolution fluorescence imaging of HeLa cells**. The super-resolution fluorescence image of actin filaments was taken with 3D-SIM and shown in yellow color. Raman intensities at 750, 1680, and 2850 cm^−1^ are mapped, representing the distributions of cytochromes (green), protein (blue), and lipid (red), respectively

Time-deterministic cryofixation is also useful for multimodal studies because switching between different modalities does not cause a time discrepancy between modes and provides the same precise snapshot in time, even when using modalities with different temporal resolutions, such as fluorescence and Raman microscopy (Fig. S24, “Materials and Methods”). Here, we demonstrated this by multimodal imaging of the same state of HeLa cells using fluorescence 3D-SIM and slit-scanning Raman microscopy (Fig. 4, Fig. S25, “Materials and Methods”).

A custom-built on-stage freezing chamber equipped with a cooling block was employed, enabling rapid freezing of cellular samples and subsequently maintaining a low temperature during spontaneous Raman imaging^51^ (Fig. S24, “Materials and Methods”), which typically requires relatively long image acquisition time, such as a few tens of minutes, due to the inherently small Raman scattering cross-section of cellular intrinsic molecules.

After cryofixation of HeLa cells, a super-resolution fluorescence image of actin filaments and high-resolution Raman images of cytochrome, protein, and lipid distributions were acquired. As shown in Fig. 4, Raman microscopy clearly visualized the spatial distribution of cytochromes, proteins, and lipids in cryofixed HeLa cell. The Raman images were reconstructed using Raman signals at 750 cm^−^^1^ (porphyrin breathing of cytochromes), 1680 cm^−1^ (amide-I of protein), and 2850 cm^−1^ (symmetric CH_2_ of lipids), respectively. Actin filaments in HeLa cells were labeled with SPY555 in the same manner, as shown in Fig. 1e. The image acquisition times for fluorescence 3D-SIM and slit-scanning Raman microscopy were 0.75 s and 25 min, respectively. Our simulation confirmed that the temperature increase during Raman imaging is ~1 °C, which would not cause any notable damage to the sample (Fig. S26). Although only two microscope imaging modalities were used in this study, a wide variety of optical microscope modalities, such as autofluorescence and coherent Raman imaging techniques, can be added, allowing us to increase multiplexing of observation targets or add further orthogonal imaging data^52^.

## Discussion

We demonstrated rapid freezing of living cells under observation by optical microscopy, in which the morphological, calcium ion, and chemical dynamics of the cells were fixed. This technique also demonstrated continuous observation of the sample with a temporal resolution of 10 ms before and after the moment of cryofixation, providing accurate spatio-temporal information on cellular dynamics and conditions immediately prior to freezing. Combining this spatiotemporal information with high spatial resolution and from cryofixed samples could offer a more detailed and comprehensive understanding of biological events. The accurate and detailed data obtained with this technique could also be useful as the ground truth for machine learning approaches^53^. In this study, we also demonstrated the precise determination of time interval (± 10 ms precision) between the initiation of cellular dynamics and its cryofixation under optical microscopy observation. This was achieved using the electrically controlled cryogen injector in conjunction with the optical stimulation technique.

One of the remarkable findings of this study is that the *K*_d_ value of fluorescent Ca^2+^ probes is maintained after rapid freezing, which indicates that the molecular-level interactions in the cell can be frozen without passing through a thermal equilibrium state. Therefore, our results suggest that rapid freezing can stop chemical reactions and can be effective for the selection of and study of chemical dynamics at a precise immobilization timepoint. As shown by the fixation of the 3D Ca^2+^ distribution, this technique can also be extended to fix the activities of other ions, including those for membrane potential and signaling molecules. This technique can be used to fix states of the cell that relate to otherwise intangible information such as pH, redox state, and temperature, which can be detected using functional fluorescent probes^1–3^.

In this study, we also observed that change in the optical properties of fluorescent probes under cryogenic conditions varies depending on the type of probes and their surrounding environments. This variability makes it important to confirm the optical properties in advances and to conduct careful analysis of observation results. For example, the difference of fluorescence intensity increases between the nucleus and cytoplasm regions after cryofixation, which was observed in the experiments for cryofixation of Ca^2+^ wave propagating in cells (Figs. 1b, S6), might be attributed to differences in surrounding environment of Fluo-4. Previous reports indicated that the optical property of negative charged Ca^2+^ indicators, including Fluo-4, is different in the nucleus and cytoplasm in living conditions^54,55^. This fact indicates the need for careful caution when analyzing spatial intensity distributions within cells. Additionally, similar caution may also be required when analyzing samples labeled with multiple kinds of fluorescent probes, as change of the optical properties of each probe could be different, potentially affecting the interpretation of results. However, it is important to note that such caution is not exclusive to cryogenic conditions; it is always necessary when using fluorescent probes, as their optical properties can be influenced by various surrounding conditions even at room temperatures, as mentioned above.

As demonstrated by cryo-TEM observations, simply pouring the liquid cryogen onto samples is effective for rapidly freezing samples without causing severe ice crystal formation. Our experiments confirmed that, even without the use of cryoprotectants, no crystalline ice was observed in two of three lamellae, each containing a cell and buffer solution (Fig. S4). Although small ice crystals were observed within cells in the remaining cellular lamella, approximately 93% of the total area was vitrified, and crystal sizes were comparable to those seen in samples frozen by plunge freezing. As with plunge freezing and high-pressure freezing, the addition of cryoprotectant, such as glycerol, may further reduce the formation of crystalline ice within cells^56^. This approach may be preferable when applying our method to higher spatial resolution techniques, such as SMLM^7–9^, STED microscopy^10^, minimum photon flux (MINFLUX) microscopy^11^, correlative light and electron microscopy (CLEM), and computational approaches^57–59^. Future developments in accurate buffer volume control devices could help to improve cooling conditions, potentially reducing ice crystal formation even without the use of cryoprotectants. In addition, while technically challenging both in implementation on an optical microscope stage and in achieving high cooling rate, high-pressure freezing techniques^60,61^ as an alternative to the current freezing method could enhance the preservation quality of cellular structures and molecular distributions by effectively suppressing ice crystal formation, enabling more precise high-resolution imaging.

Although the main target of this research is time-deterministic fixation for optical microscopy, the open-top design also facilitates sample processing after freezing. For example, subsequent freeze substitution, staining, and resin embedding^62^ are all enabled by this design. Developing a device that can exchange liquid cryogen, which is maintained on a sample after rapid freezing, with freeze substitution media while maintaining the sample at cryogenic temperatures remains essential. The open-top and simple construction of our sample freezing chamber is expected to be advantageous for this development. Combination with electron microscopy also holds many potential advantages; however, depending on the application, it is necessary to investigate the freezing characteristics and the capability to preserve ultrastructural integrity.

Analysis of cellular dynamics and related functions using the rapid freezing techniques under optical microscopy observations still remains a largely unexplored field with many challenges and points requiring careful interpretation. However, as demonstrated and discussed in this paper, the potential benefits of this technique are significant. Compared with chemical fixation, cryofixation produces fewer morphological and chemical artifacts^15,18,19^. In addition, chemical environments such as ion distributions, pH, and redox conditions may also be fixed, allowing a more detailed investigation of chemical responses that govern biological phenomena. The rapid freezing can even immobilize the states of both endogenous and exogenous molecules, such as fluorescent indicators, providing snapshots of molecular behavior at a moment of interest. Therefore, by exploiting the rapid freezing techniques under optical microscopy observations, detailed visualization of chemically unfixable targets, such as ions or other signaling messengers, as well as other observation targets at specific moments of cellular dynamics would become possible, allowing for more detailed understanding of their intracellular distribution and interaction without being limited by short exposure times needed for traditional time-resolved imaging. This advantage is also significantly beneficial for enhancing the imaging performance of super-resolution fluorescence microscopy^7–12^ which can benefit greatly from relaxing constraints on acquisition times. The concept and design of fluorescent probes can also be altered when considering the usage and optimization of their performances under cryogenic conditions. This technique is also useful for improving the detection sensitivities of optical techniques that require the detection of low-light signals, such as Raman microscopy. Cryogenic Raman imaging using Raman tags can be used to observe low concentrations of small molecules in live cells that are difficult to observe using fluorescence labeling^63,64^. Further improvement in SNR and detection sensitivities could be achieved if an immersion objective lens with a high numerical aperture is used, which allows us to improve the spatial resolution^65–67^. Moreover, the implementation of adaptive optics can further optimize the spatial resolution and signal levels.

Although various challenges still remain in our technique as discussed above, the techniques presented in this paper demonstrated a significant advancement in terms of ability for rapid and time-deterministic immobilization of cellular dynamics under optical microscopy observation. The simplicity and user-friendliness of on-stage rapid freezing technique facilitate practical usage of cryogenic optical microscopy, broadening its application across a wide range of research fields relying on optical microscopy. This advancement also has the potential to stimulate further development of various techniques related to cryogenic optical imaging.

## Materials and methods

The materials and methods are described below, with further experimental details provided in the Supplementary Information.

### On-stage freezing chamber

The sample freezing chamber consisted of three parts: top, middle, and bottom mounts, as shown in Fig. 1a. A sample coverslip was placed in the indented area of the bottom mount and held by pressing it down from above. The top mount was equipped with two hinges. After a sample is mounted, it can then be cryofixed by applying liquid cryogen from the upper side, and the sample freezing chamber allows the storage of liquid cryogen during observation. The area between the sample coverslip and the objective lens was filled with nitrogen gas, which was kept in a chamber with a plastic sheet and an objective-lens-sized hole in the center to prevent frost condensation due to low temperatures. This simple design facilitates easy handling of this sample freezing chamber, significantly reducing the time required to exchange a sample to approximately 30-60 s by using spare mounts. This sample freezing chamber can be attached to any type of standard inverted microscope stage using appropriate adapters.

A sample (cells on a 10 × 10 mm, 18 × 18 mm, or 25 mm diameter quartz coverslip (thickness of 0.17 ± 0.02 mm)) was removed from the cell culture medium or a buffer solution in a cell culture dish, and the liquid on the bottom side of the coverslip was carefully wiped off with paper such as lens cleaning paper with ethanol. Immediately thereafter, the sample was mounted in an on-stage freezing chamber, and a buffer solution, such as Hank’s balanced salt solution (HBSS) or phosphate-buffered saline (PBS), was added to prevent the sample from drying out while it was being prepared for measurement.

The experimental data shown in this paper were obtained using a Nikon Ti2-E microscope. After completing the measurement preparation and activating the axial autofocus system of the Ti2-E microscope, the buffer solution was aspirated using a pipette. Under an optical microscope, liquid cryogen was applied to freeze a sample at any given time, such as when Ca^2+^ waves or Ca^2+^ transients occur.

### Liquid cryogen

For our experiments, a mixture of liquid isopentane and propane (temperature: about −185 °C, ratio of isopentane to propane: 1:2–1:3) was mainly used. By mixing isopentane and propane, the freezing point is lowered, allowing us to handle the cryogen more easily while it remains in liquid even after more than 30 min of cooling with liquid nitrogen (LN_2_)^68^. Only for multimodal imaging (Fig. 4), liquid propane (temperature: about −185 °C) was used.

### Optical microscopy setups

Four optical microscopy modalities were employed in this study: a widefield fluorescence microscope, a structured illumination microscopy (SIM) system, a hyperspectral slit-scanning fluorescence microscope, and a multimodal system combining SIM with slit-scanning Raman microscopy. The base platform was a Nikon Ti2-E inverted fluorescence microscope equipped with a mercury lamp and sCMOS camera (Hamamatsu Photonics, ORCA Flash4.0 V3). An axial autofocus system was activated to maintain image focus during observations.

For SIM imaging, a laser excitation path and a reflective-type spatial light modulator were added to the widefield microscope to generate structured illumination patterns on samples (Fig. S10). To acquire a single X-Y image using 3D-SIM microscopy, 15 images of a sample were acquired using structured illumination at three different illumination angles with five phase shifts per angle, which were then reconstructed into a SIM image. For 3D volumetric SIM imaging, this process was repeated at different Z-positions by changing the Z-direction offset position of the autofocus function of a Nikon Ti2-E microscope, and the resultant image stack was used to construct a 3D-SIM volumetric image. Fluo-4 (AAT Bioquest, 20550 or Chemical Dojin, 342-90961) and the live-cell F-actin probe SPY-555-actin (Spirochrome, SC202) were excited using 488-nm and 561-nm lasers, respectively. Fluorescence signals were detected using the sCMOS camera (Hamamatsu Photonics, ORCA Flash4.0 V3), which was also used for widefield imaging.

For slit-scanning hyperspectral fluorescence imaging, the widefield microscope was modified to include a 405-nm laser excitation path to generate a line illumination, along with a spectrometer equipped with an electron multiplying charged-coupled device (EMCCD) camera (Princeton Instruments, Pro-EM:1024) (Fig. S19). Fluorescence spectra were acquired via line-illumination and slit-confocal detection and were subsequently used to reconstruct fluorescence images.

The multimodal system extended the SIM microscope with an additional optical setup for a slit-scanning Raman microscopy (Fig. S24). The setup included a 532 nm laser excitation path for line illumination and a spectrometer (Bunkoukeiki, MK-300) equipped with a cooled CCD camera (Princeton Instruments, PIXIS:400BR). After SIM imaging, Raman spectra from a sample were recorded and used to reconstruct Raman images.

### Cellular sample preparations

Neonatal rat cardiomyocytes were cultured on gelatin-coated coverslips, whereas HeLa cells and COS-7 cells were cultured on uncoated coverslips. The culture medium was Dulbecco’s modified Eagle’s medium containing 10% fetal bovine serum and 1% PSG antibiotic mix (100 U mL^−1^ penicillin, 100 μg mL^−1^ streptomycin, 2 mM l-glutamine).

For Ca^2+^ imaging of neonatal rat cardiomyocytes, the cells were washed three times with HBSS, immersed in Fluo-4 (AAT Bioquest, 20550 or Chemical Dojin, 342-90961) staining solution, and then washed again three times prior to imaging. For dual color imaging of Ca^2+^ and F-actin, neonatal rat cardiomyocytes were pre-stained with the live-cell F-actin probe SPY-555-actin (Spirochrome, SC202), followed by Fluo-4 staining. To perform Ca^2+^ imaging with a caged calcium compound (Tocris, DMNPE-4 AM-caged calcium, 5948), the cells were loaded with the mixture of Fluo-4 and the caged calcium compound following the same protocol as Fluo-4 staining. Ratiometric Ca^2+^ imaging of HeLa cells was conducted with stably expressing YC3.60 in the cytosol.

For organelle observation, mitochondria in HeLa cells were labeled by using pcDNA3 encoding DsRed. HeLa cells were imaged 1 day after transfection. To observe lysosomes in COS-7 cells, the cells were incubated with the live-cell lysosome probe LysoTracker Red NDN-99 (Thermo Fisher Scientific, L7528), then washed three times before imaging.

## Supplementary information

Supplemental material

VideoS1

VideoS2

VideoS3

VideoS4

VideoS5

VideoS6

## Data Availability

All original data used in this manuscript are available from the corresponding authors upon reasonable request.
