# Supplementary material for: Time-deterministic cryo-optical microscopy
Source: Light Sci Appl. 2025 Aug 23;14:275. doi: 10.1038/s41377-025-01941-8 (PMC12373960; doi:10.1038/s41377-025-01941-8)
Supplement: Supplementary file 1 — Supplemental material [file 41377_2025_1941_MOESM1_ESM.pdf]

Supplementary Information for

Time-deterministic cryo-optical microscopy

Kosuke Tsuji, Masahito Yamanaka, Yasuaki Kumamoto, Shoko Tamura, Wakana Miyamura, Toshiki Kubo, Kenta Mizushima, Kakeru Kono, Hanae Hirano, Momoko Shiozaki, Xiaowei Zhao, Heqi Xi, Kazunori Sugiura, Shun-ichi Fukushima, Takumi Kunimoto, Yoshino Tanabe, Kentaro Nishida, Kentaro Mochizuki, Yoshinori Harada, Nicholas I. Smith, Rainer Heintzmann, Zhiheng Yu, Meng C. Wang, Takeharu Nagai, Hideo Tanaka, Katsumasa Fujita

Corresponding authors
Katsumasa Fujita
E-mail: fujita@ap.eng.osaka-u.ac.jp

Masahito Yamanaka
E-mail: yamanaka@ap.eng.osaka-u.ac.jp

This supporting information includes:

Supporting text
Figures S1 to S28
Legends for Videos S1 to S6
References

Other supporting materials for this manuscript include the following:

Videos S1 to S6

Supporting Information Text

Materials and Methods

On-stage freezing chamber: Fabrication

The top, middle, and bottom mounts were fabricated using commercially available 3D printers. The top mount was fabricated using polylactic acid (PLA) (UltiMaker, Tough PLA) by a fused-filament fabrication-type 3D printer (UltiMaker, UltiMaker S5). The PLA material does not break at -196 °C and has low thermal conductivity (0.13 W m^{-1} K^{-1}). The top mount was designed as a hollow structure with 90% air (0.0257 W m^{-1} K^{-1} at 20 °C) to improve the thermal insulation between the top mount and surroundings. The middle and bottom mounts are in direct contact with a sample coverslip; therefore, the surface in contact with the sample coverslip must be flat and smooth to prevent the buffer solution of the sample from leaking from the mount. For this reason, these middle and bottom mounts were fabricated using a stereolithography-type 3D printer (Formlabs, Form3), which enables more precise and smoother fabrication than fused filament fabrication-type 3D printers. Rigid 10K resin (Formlabs) was used as a stereolithography-type 3D printer to produce the middle and bottom mounts.

Liquid cryogen: Preparation and cooling performance evaluation

Liquid cryogens were prepared using a semiautomated custom-built cryogen preparation apparatus (Cryovac, CV-Z-LTMIX100). To prepare the mixture of liquid isopentane and propane, liquid isopentane was poured into a glass beaker and placed in the preparation apparatus. The liquid isopentane in the beaker was cooled with LN_2, and propane gas was introduced into the cooled liquid isopentane and liquefied in the liquid isopentane. Throughout the liquefaction process of propane gas, the mixture was continuously stirred. When the preparation finished, the beaker was taken out of the apparatus, and the mixture was cooled down to about -185 °C with LN_2 and kept until needed for the experiments.

To prepare liquid propane, an empty glass beaker was placed in the apparatus and cooled with LN_2. Propane gas was blown onto the inner surface of the beaker for liquefaction. Then, the liquid propane was cooled down to about -185 °C with LN_2 in the same way as in the case of a mixture of liquid isopentane and propane.

Using the cryogen, a cooling rate was estimated with pure water placed on a coverslip mounted in this freezing chamber. In this measurement, a thermocouple with a height of 25 μm (ANBE SMT Co., KFT-25-100-100) was placed on a coverslip, and approximately 0.2 μL pure water was dropped onto the thermocouple. The water thickness was estimated to be approximately 40 μm from the observed spread size of water on a coverslip. Liquid cryogen was then applied onto the water, and the resulting cooling rate was recorded. A cooling rate of 10500 °C s^{-1} was observed from room temperature (15 °C) down to -100 °C (Fig. S28). In actual cell-freezing experiments in this study, the thickness of residual buffer solution was estimated to be 6.7 ± 2.5 μm (including cell thickness), as shown in Fig. S1, suggesting that higher cooling rate was likely achieved in these conditions.

We also confirmed the cooling rate when the liquid cryogen is directly applied onto the thermocouple (25 μm height, ANBE SMT Co., KFT-25-100-100). The cooling rates from room temperature to approximately -180 °C were 48500 °C s^{-1} when poured from a beaker, and 98500 °C s^{-1} when injected using the cryogen injector (Cryogen injector: Fig. S16, the data of cooling rate: Fig. S28). These rates are approximately 1.3–2.5 times lower than those reported for plunge freezing with a bare thermocouple (Ref. 1), likely due to the differences in cooling geometry: our on-stage freezing chamber applies the liquid cryogen from above, enabling unidirectional cooling, whereas plunge freezing immerses the thermocouple into the cryogen, allowing multidirectional cooling.

Considering the measured cooling rate and the results of cryo-TEM imaging shown in Fig. S4, the use of the mixed cryogen for our on-stage freezing can provide a freezing condition applicable for cryofixation in optical microscopy.

Freezing cells for cryo-TEM and cryo-ET

HeLa cells were cultured on a TEM grid coated with a layer of continuous carbon film (Electron Microscopy Sciences, Carbon Film 150 Mesh, Au grid bar), which was placed in a 35-mm dish at 37 °C with 5% CO_2. Prior to freezing, the nuclei were stained with Hoechst (1:1000 dilution in PBS) for 5 min at 20 °C to help find the region of interest in cryo-FIB/SEM. Excess PBS was gently blotted using a Kimwipe while holding the grid with tweezers. Immediately after blotting, the grid was placed at the bottom of a cylindrical Styrofoam container, and 30 mL of liquid cryogen (isopentane/propane, -186 °C) was poured onto it. The grid was kept in the liquid cryogen for 1-2 min. The grid was then transferred into a grid box in LN_2.

Cryo-FIB milling

To prepare thin, electron-transparent lamellae of HeLa cells, grids were mounted into Autogrids (Thermo Fisher Scientific). These Autogrids were then loaded into an Aquilos 2 cryo focused ion beam (FIB) / scanning electron microscope (SEM) system (Thermo Fisher Scientific). Throughout the FIB milling process, the samples were maintained at -180 °C by the cryo-stage.

An overview image of the EM grid was acquired by SEM, with the following parameters: an acceleration voltage of 2 kV, beam current of 13 pA, nominal magnification of 276×, pixel size of 163 nm, and a dwell time of 1 μs. Subsequently, a thin metallic platinum layer was sputtered onto the grid at 30 mA, 10 Pa, for 15 s. Using the gas injection system, gaseous trimethyl (methylcyclopentadienyl) platinum was deposited over the grid for 75 s. To navigate cells on the EM grids, Hoechst fluorescent images were acquired using integrated Fluorescence Light Microscope (iFLM) equipped with Aquilos 2 cryoFIB-SEM with the following parameters: filter of 385 nm, light source intensity at 5%, and an exposure time of 400 ms. Reflection images were obtained with filter of 385 nm, light source intensity at 1%, and an exposure time of 0.5 ms. The acquired images were imported into Maps3.28 software and aligned with SEM data to determine the locations of cells.

Lamellae were milled at the milling angle of 20° with the FIB operating at 30 kV. The beam current was progressively reduced from 1 nA to 50 pA using AutoTEM2.4, resulting in a final lamella thickness of

approximately 200 nm. Manual polishing was performed to further refine the lamellae to a thickness of around 180 nm, by a cleaning cross section pattern with 50 pA beam current. The prepared grids were stored in LN_2 until TEM data collection.

Cryo-TEM and cryo-ET

Cryo-EM imaging was performed on a Titan Krios (Thermo Fisher Scientific) equipped with a high-brightness field emission gun (xFEG), a spherical aberration corrector, a BioContinuum HD energy filter (Gatan), and a K3 direct detector (Gatan). Lamella survey maps were acquired at a nominal magnification 2250× with a defocus of 100 µm. A 70 µm objective aperture and a 20-eV energy slit were used for the survey map generation and 20 eV energy slit. Tomographic tilt-series were collected using a dose symmetric scheme over a tilt range from -48° to +48° with a 3° increment and -20° starting angle to compensate for the 20° milling angle of lamella. For each tilt angle, a movie was recorded at a nominal magnification of 19500×, corresponding to a calibrated pixel size of 3.608 Å in CDS (correlated double sampling) counted mode. The total dose for a tilt series was 150 e^{-} $Å^{-2}$ and the defocus ranged from -2 µm to -4 µm. A 6-eV energy slit and a 70 µm objective aperture were used for the tilt series collection. Semiautomated data collection was set up in SerialEM with customized scripts.

Sample preparations and staining

a) Neonatal rat cardiomyocyte

All animal experiments described in this study were conducted in accordance with the *Guide for the Care and Use of Laboratory Animals* (8th edition, National Academies Press, Washington DC, 2011) following the approval of the Animal Research Committee at the Kyoto Prefectural University of Medicine (approval No: M2022-238, M2023-231, M2024-209). Primary cultures of neonatal rat cardiac myocytes were prepared as previously described with some modifications[2,3]. Briefly, 2- or 3-day-old Wistar rats (Japan SLC, Inc.) were anesthetized using intraperitoneal injection with 0.1 mg kg^{-1} of medetomidine, 3.0 mg kg^{-1} of midazolam, and 5.0 mg kg^{-1} of butorphanol, and the hearts were removed and placed in Ca^{2+}- and Mg^{2+}-free PBS, which was prepared by dissolving one PBS tablet (Takara, T9181) in 1000 mL of deionized distilled water. The aorta was discarded, and the atria and ventricles were minced under aseptic conditions. The small pieces were enzymatically digested three times for 10 min each with 10 mL of PBS containing 0.2% type II collagenase (Worthington, CLS2) at 37 °C. Cell suspensions from each digestion were pooled, centrifuged at 800 rpm for 5 min, and resuspended in Dulbecco's modified Eagle's medium (DMEM) (FUJIFILM Wako Pure Chemical, 043-30085) supplemented with 10% fetal bovine serum (FBS) (Nichirei biosciences inc., 175012) and 1% PSG antibiotic mix (100 U mL^{-1} penicillin, 100 µg mL^{-1} streptomycin, 2 mM l-glutamine) (FUJIFILM Wako Pure Chemical, 161-23201). To eliminate non-myocyte cells from the preparation, cells were pre-plated twice in a 100-mm culture dish for 45 min at 37 °C in a humidified atmosphere containing 5% CO_2-95% room air. After the pre-plating step, the cell suspension containing myocytes was collected and plated on gelatin-coated

coverslips in DMEM containing 10% FBS and 1% PSG. 24 h after plating, cells were washed with fresh DMEM for experiments.

The Ca^{2+} indicator Fluo-4 AM (AAT Bioquest, 20550 or Chemical Dojin, 342-90961) was used for Ca^{2+} imaging, as shown in Fig. 1B, 1E, 1F, 2B-2D, 3A, and S6. To prepare the staining solution, solid Fluo-4 AM was dissolved in dimethyl sulfoxide (DMSO) (FUJIFILM Wako Pure Chemical, 048-32811), and its concentration was adjusted to 1 mM. The Fluo-4 AM solution was mixed with nonionic detergent Pluonic® F-127 (Biotium, 59004), and then diluted to 1 μM with HBSS (FUJIFILM Wako Pure Chemical, 082-08961) to prepare a staining solution. Neonatal rat cardiomyocytes were washed three times with HBSS, immersed in Fluo-4 staining solution for 15 min, and washed three times with HBSS. In the experiments shown in Fig. S6, neonatal rat cardiomyocytes were immersed with a HEPES buffered Tyrode solution with a Ca^{2+} concentration of 14.4 mM for frequent induction of Ca^{2+} waves.

The live-cell F-actin probe SPY-555-actin (Spirochrome, SC202) was used to image actin filaments (Fig. 1E). Solid SPY-555 was dissolved in 50 μL of DMSO (FUJIFILM Wako Pure Chemical, 048-32811) to prepare a stock solution, and then the stock solution was diluted 1000-fold with the cell culture medium to prepare a staining solution. Cardiomyocytes were immersed in the staining solution for 1 h and washed three times with HBSS.

To perform Ca^{2+} imaging with a caged calcium compound (Tocris, DMNPE-4 AM-caged calcium, 5948), both the caged calcium compound and Fluo-4 were loaded into cardiomyocytes. The caged calcium compound was dissolved in DMSO at a concentration of 100 mM. The caged calcium compound solution was mixed with a mixture of Fluo-4 and the nonionic detergent Pluonic® F-127 (Biotium, 59004). The mixture was diluted with HBSS to prepare the staining solution. The concentrations of Fluo-4 and caged calcium compounds in the staining solutions were 1 and 10 μM, respectively. Cardiomyocytes were stained in the same manner as for Fluo-4 staining.

b) Fluo-4 and Yellow Cameleon 3.60 (YC3.60) in calcium calibration buffer solution

A calcium calibration buffer solution kit (Thermo Fisher Scientific, C3008MP) was used to prepare free Ca^{2+} buffer solutions with various Ca^{2+} concentrations. To measure K_d of Fluo-4, Fluo-4 pentapotassium salt (ATT Bioquest, 20555) was dissolved in a buffer solution containing 10 mM EGTA, 100 mM KCl, and 30 mM MOPS (Thermo Fisher Scientific, C3008MP) and was mixed with 39 μM free Ca^{2+} buffer solution (Thermo Fisher Scientific, C3008MP) to prepare the free Ca^{2+} buffer solutions with different Ca^{2+} concentrations. The concentration of Fluo-4 was 5 μM. As in the case of Fluo-4, YC3.60 was dispersed in a free Ca^{2+} buffer solution for the measurement of K_d. The concentration of YC3.60 was 3.6 μM.

c) HeLa cell

HeLa cells (JCRB Cell Bank, JCRB9004) were grown in DMEM (FUJIFILM Wako Pure Chemical, 043-30085) supplemented with 10% fetal bovine serum (FBS) (BioWest, S1820) and 1% PSG antibiotic mix (100

U mL^{-1} penicillin, 100 μg mL^{-1} streptomycin, 2 mM l-glutamine) (gibco, 10378-016) and cultured on coverslips under the conditions of 5% CO_2 and 37 °C.

To observe the mitochondria in HeLa cells (Fig. 1G), pcDNA3 encoding DsRed, which fused CoxVIII signal peptides, was introduced into HeLa cells, using cationic lipid-mediated transfection. For transfection, HeLa cells were grown to 50-60% of confluence in 6-well plates. Then, reduced-serum medium Opti-MEM (Thermo Fisher Scientific, 31985062) containing 1 μg fluorescent protein plasmid DNA and cationic lipid-based transfection reagents (1μL Lipofectamine 3000 reagent and 5 μL P3000 reagent (Thermo Fisher Scientific, L3000008)) were added to each well, and HeLa cells were incubated for 1 day in cell culture.

For multimodal SIM/Raman imaging, the HeLa cells were cultured on coverslips and stained with SPY-555-actin (Spirochrome, SC202). The staining condition was the same as that described in the section on neonatal rat cardiomyocytes.

To perform ratiometric Ca^{2+} imaging (Fig. 3B), HeLa cells stably expressing YC3.60 in the cytosol were used. HeLa S3 (RIKEN BRC) cells, transformed using YC3.60/pcDNA3, were dilution cultured in Dulbecco's modified eagle's medium (WAKO, 041-29775) with 10% fetal bovine serum (BioWest) in the presence of 0.5 mg mL^{-1} genethicin (gibco,10131-035). The colony derived from a single cell was collected and cultured again in Dulbecco's modified eagle's medium to obtain a stable expression strain.

d) COS-7 cell

COS-7 cells (JCRB Cell Bank, JCRB9127) were grown in low-glucose DMEM (FUJIFILM Wako Pure Chemical, 041-29775) supplemented with 10% fetal bovine serum (FBS) (BioWest, S1820) and 1% PSG antibiotic mix (100 U mL^{-1} penicillin, 100 μg mL^{-1} streptomycin, 2 mM l-glutamine) (gibco, 10378-016) and cultured on coverslips under 5% CO_2 and 37 °C.

The live-cell lysosome probe LysoTracker Red NDN-99 (Thermo Fisher Scientific, L7528) was used to observe the distribution and dynamics of lysosomes. Solid LysoTracker Red NDN-99 was dissolved in DMSO and diluted with cell culture medium to prepare a 75 nM staining solution. COS-7 cells were immersed in the staining solution for 2 h and washed three times with HBSS.

For evaluating the morphological changes by cryofixation, COS-7 cells were fixed with 4% paraformaldehyde (FUJIFILM Wako Pure Chemical, 161-20141), permeabilized with 0.1% Triton X-100 (Kishida Chemical, 020-81155), and blocked with 4% bovine serum albumin (Sigma-Aldrich, A2153-50G). The actin filaments were then labeled using 500 nM Phalloidin-ATTO488 (Sigma-Aldrich, 49409-10NMOL) to visualize its distribution.

e) Protein preparation of recombinant YC3.60, Venus, and ECFP.

To yield the recombinant protein, *E. coli* [JM109 (DE3) (Promega, P9801)] was transformed using $pRSET_B$ vectors (Invitrogen, V35120) encoding YC3.60, ECFP and Venus, and cultured in 200 mL liquid LB medium at 23 °C for 60-72 h. *E. coli* was collected by centrifugation, resuspended in buffer containing 50 mM Tris-HCl (pH 8.0) (MP Biomedicals, 02103130-CF) and 20 mM imidazole (Wako, 099-00013), and crushed using

a French press. The supernatant of the centrifuged bacterial crushing solution was adsorbed onto Ni-NTA resin (Qiagen, 30250) in an open column, washed with buffer for resuspension, and eluted with buffer containing 50 mM Tris- HCl (pH 8.0) 250 mM imidazole. Finally, the buffer was replaced with 10 mM EGTA (DOJINDO, G002) in 100 mM KCl (WAKO, 7447-40-7), 30 mM MOPS (DOJINDO, GB13) (pH 7.2) using PD-10 column (Cytiva).

Observation and cryofixation of Fluo-4 loaded neonatal rat cardiomyocytes

The Fluo-4 loaded neonatal rat cardiomyocytes were rapidly frozen under microscopic observation with a conventional inverted widefield fluorescence microscope (Nikon, Ti2-E) equipped with a mercury lamp. The Ca^{2+} wave propagation stopped at the image frame at 0 ms, as shown in Fig. 1B, 1C, S6, and S8, and it was considered that the liquid cryogen contacted the sample at the image. The contact timing of liquid cryogen on the sample was determined by finding the image frame in the time-series images where the fluorescence intensity suddenly increased over the entire area of the image. This is because the fluorescence intensities of fluorescent dyes are known to increase at low temperatures owing to an increase in their quantum yields[4,5] (Ref. 32 in the main text). In this case, the fluorescence intensity of Fluo-4 increased over the entire area of the image frame at 0 ms, and the enhancement factor was 1.18 compared to that in the immediate previous image frame.

For visualization, the fluorescence intensity of each image was normalized to the fluorescence intensity of the selected area in the observed cell. As the fluorescence intensity was enhanced under cryogenic conditions, we applied normalization for the fluorescence images separately before and after cryofixation. First, the background signal values before and after cryofixation were obtained from an area where there were no cardiomyocytes in each image, and then subtracted from each image. To normalize the fluorescence images before cryofixation, we chose one area in the fluorescence image of an observed cardiomyocyte just before cryofixation and took the mean intensity value. Fluorescence images before cryofixation were divided by the mean intensity value. To normalize the fluorescence images under cryogenic conditions, mean intensity values at the same position in each image as that before cryofixation were used. In contrast to normalization of the images before cryofixation, each fluorescence image under cryogenic condition was divided by the mean intensity values taken in each fluorescence image. This was performed to compensate for the change in fluorescence intensity caused by temperature fluctuation or increase after cryofixation. We noticed that the sample position drifted slightly after cryofixation, probably because of temperature changes. Therefore, we compensated for the drift motion by estimating the displacement from the cross-correlation of the phase components in the Fourier transformation of the images[6].

The excitation and detection wavelengths were 464-499 nm (Semrock, FF01-482/35-25) and 516-556 nm (Semrock, FF01-536/40-25), respectively. The sample was observed with a 0.7 NA dry objective lens (Nikon, CFI S Plan Fluor ELWD 60XC), and fluorescence images were acquired using an sCMOS camera (Hamamatsu Photonics, ORCA Flash4.0 V3). The autofocus system of the Nikon microscope was activated during the observations; the exposure time was 10 ms and the frame rate was 100 frames s^{-1}. For the

experiment presented in Fig. S6, no trehalose was added to the buffer solution. In the experiment shown in Fig. 1B, 2C and 2D, we added trehalose (FUJIFILM Wako Pure Chemical, 204-18451) to a buffer solution as a cryoprotectant. The concentration of trehalose was 200 mM.

In a similar manner as in Fig. 1B, the data shown in Fig**.** 2C and 2D were acquired and the fluorescence intensity was normalized.

Dissociation constant measurement

The dissociation constants (K_d) of calcium indicator Fluo-4 were measured under 20 °C (room temperature) and -180 °C (cryofixed). In the measurements under 20 °C (room temperature) and -180 °C (cryofixed), we repeated the following procedure: 1) we measured fluorescence signals of a Fluo-4 solution with a certain Ca^{2+} concentration under 20 °C, 2) froze it rapidly with a liquid cryogen and measured fluorescence signals under -180 °C, and then 3) changed the Fluo-4 solution to one with different Ca^{2+} concentration. The fluorescence signals were measured at 10 different observation positions in the sample, and then the averaged values and the standard deviations were calculated. The error bars represent the standard deviations. Fluorescence intensity was plotted against Ca^{2+} concentration on a logarithmic x-axis. The K_d values were obtained by fitting the measured data with a sigmoidal function[7, 8].

Fluorescence signals were measured with a conventional widefield inverted fluorescence microscope (Nikon, Ti2-E) equipped with a mercury lamp. Fluorescence signals were collected with a 0.7 NA dry objective lens (Nikon, CFI S Plan Fluor ELWD 60XC) and acquired with a spectrophotometer (Princeton Instruments, Acton SP2500) equipped with an electron multiplying charged-couple device (EMCCD) camera (Andor, iXon Ultra 888). The excitation wavelength band was 464-499 nm (Semrock, FF01-482/35-25) and the fluorescence signals were detected in a wavelength region above 529 nm (Semrock, BLP01-514R-25).

In the same way as for the case of Fluo-4, fluorescence spectra of YC3.60 were acquired with a spectrophotometer equipped with an EMCCD camera (Andor, iXon Ultra 888) under different Ca^{2+} concentrations. To calculate the fluorescence ratio, we used the same wavelength regions as those used for reconstructing the fluorescence ratio images in Fig. 3B. The excitation wavelength band was 400-410 nm (Semrock, FF01-405/10-25). The fluorescence signals were detected in a wavelength region above 461 nm (Semrock, LP03-458RU-25). A 0.7 NA dry objective lens (Nikon, CFI S Plan Fluor ELWD 60XC) was used for this observation.

Super-resolution imaging of cryofixed neonatal rat cardiomyocytes using 3D-SIM

After the sample was cryofixed during observation using a widefield fluorescence microscope, images were obtained by 3D-SIM under cryogenic conditions (Fig. S10). Because the actin probe SPY555 allowed us to label F-actin, the gaps between actin filaments in the sarcomeres of neonatal rat cardiomyocytes were also more clearly observed using 3D-SIM (Ref. 12 of the main text). In this observation, the exposure time was 25 ms for structured illumination at each angle and phase, and 15 raw images with three angles and five phases per angle were acquired. Trehalose was added to the buffer solution as a cryoprotectant for the

experiment in Fig. 1E, but not for the experiment in Fig. 1F. Initially, trehalose, one commonly used cryoprotectant for electron microscopy observation of cryofixed samples, was added to mitigate potential structural deformation caused by cryofixation. However, during this study, optical microscopy observation did not show noticeable differences in deformation between samples with or without trehalose (Fig. S27). Therefore, in several experiments, including Fig. 1F, trehalose was not added.

In Fig. 1F, Ca^{2+} wave propagation in neonatal rat cardiomyocytes was stopped by cryofixation under a microscopic observation with a conventional inverted widefield fluorescence microscope (Nikon, Ti2-E) equipped with a mercury lamp, and then 3D imaging with 3D-SIM was performed. To acquire a 3D image, the autofocus system of the Nikon microscope was activated during observation, and axial scanning was performed by changing the Z-direction offset position of the autofocus system in steps using homemade software. The step size along the Z direction was 285 nm. The other experimental parameters were identical to those shown in Fig. 1E. In Video S2, the fluorescence intensities of each image were normalized for visualization in the same manner as shown in Fig. 1B.

Image acquisition and reconstruction of SIM images were performed using homemade software written in C# and MATLAB. The reconstruction software allows us to perform pre-processing before reconstructing a SIM image from raw images. The pre-processing process involves normalizing fluorescence intensities among raw images and correcting small sample drifts through phase correlation. For SIM image reconstruction, a pre-processed image set is spatially Fourier transformed, and the high spatial frequency components are separated and reassigned in the spatial frequency space through a matrix-based unmixing process. If there is a mismatch between an experimental result and a theoretical estimate of reconstruction parameters, the mismatch produces artifacts in a reconstructed image. To reduce the artifacts, this software also allows us to optimize the reconstruction parameters, such as the phases and frequencies of structured illumination[9]. A Wiener filter and weighting averaging were also applied to each frequency component to reduce the Poisson noise in the frequency space and enhance the high-frequency components achieved by structured illumination[10]. Finally, a super-resolution image was reconstructed by inverse Fourier transforming the resultant frequency distributions.

Observation and cryofixation of mitochondria in HeLa cells and lysosomes in COS-7 cells

HeLa cells expressing DsRed in mitochondria were observed with a conventional inverted widefield fluorescence microscope equipped with a mercury lamp. As shown in Fig. 1G, the dynamic motion of the mitochondria was immediately stopped when the liquid cryogen was introduced for cryofixation. The excitation and detection wavelength bands were 527-552 nm (Nikon, excitation filter of TRITC filter cube set) and 577-632 nm (Nikon, emission filter of TRITC filter cube set), respectively. The sample was observed with a 0.7 NA dry objective lens (Nikon, CFI S Plan Fluor ELWD 60XC) and fluorescence images were acquired with an sCMOS camera (Hamamatsu Photonics, ORCA Flash4.0 V3). The exposure time was 1 s and the frame rate was 1 frame s^{-1}. In this experiment, it is noted that the sample was cryofixed midway through the acquisition of a single image. Therefore, the subsequent frame is presented as the initial frame,

which is denoted as 0 s, under cryogenic conditions in Fig. 1G. Similar to Fig. 1B, fluorescence images were normalized for visualization purposes. Prior to normalization, background signal values were obtained from an area where there was no HeLa cell in each image, and then subtracted from each image. To normalize the fluorescence images after the background signal subtraction, we chose one bright area in the fluorescence images of an observed HeLa cell and took the mean intensity values in each image. Then, each fluorescence image was divided by each mean intensity value. This normalization procedure allowed compensation for changes in fluorescence intensity caused by factors such as photobleaching, temperature decrease due to rapid freezing, and temperature fluctuations after cryofixation. This normalization was also applied to fluorescence images of lysosomes in COS-7 cells.

COS-7 cells labeled with LysoTracker Red (Thermo Fisher Scientific, L7528) were observed using a conventional inverted wide-field fluorescence microscope equipped with a mercury lamp. As shown in this movie, the dynamic motion of the lysosomes was observed at room temperature and was immediately stopped by cryofixation. The excitation and detection wavelength bands were 560/40 nm (Nikon, excitation filter of Texas Red filter cube set) and 630/60 nm (Nikon, emission filter of Texas Red filter cube set). The sample was observed with a 0.7 NA dry objective lens (Nikon, CFI S Plan Fluor ELWD 60XC), and fluorescence images were acquired with an sCMOS camera (Hamamatsu Photonics, ORCA Flash4.0 V3), with an exposure time of 200 ms and the frame rate was 5 frames s^{-1}.

In both experiments, we added trehalose in a buffer solution as a cryoprotectant. The concentration of trehalose was 200 mM. To maintain the sample temperature at 37 °C prior to rapid freezing, we used a silicone rubber heater integrated in the bottom mount of the on-stage freezing chamber. The temperature of the buffer solution was monitored using a thermocouple.

Observation and cryofixation of Ca^{2+} wave induced by uncaging Ca^{2+} from a caged Ca^{2+} compound with UV light irradiation

Neonatal rat cardiomyocytes were loaded with Fluo-4 and caged calcium compound (Tocris, DMNPE-4 AM-caged-calcium). During widefield fluorescence observations with a mercury lamp, UV light was focused on a cardiomyocyte for 60 ms to uncage Ca^{2+} and then they were rapidly frozen at 120 ms after UV irradiation. At the time of the UV irradiation, the bright spot appeared in the fluorescence imaging as shown in the fluorescence image at -120 ms. After uncaging Ca^{2+}, the Ca^{2+} wave propagated through Ca^{2+} induced Ca^{2+} release (CICR), and then the Ca^{2+} wave was stopped during the propagation by cryofixation. In this experiment, we added trehalose in a buffer solution as a cryoprotectant. The sample drift was compensated, and the fluorescence intensities of each image were normalized for visualization purposes in the same manner as that in Fig. 1B.

We developed a cryogen injector with Cryovac Inc (Fig. S16). When utilizing the cryogen injector, a pre-prepared mixture of liquid isopentane and propane is introduced through an inlet port of the cryogen injector (Cryovac Inc., CV-22Z-LCDM-M) before conducting sample observations. Within the cryogen injector, the outer compartment of the tank containing the mixed liquid cryogen within the cryogen injector is filled with

LN_2, which cools the mixed liquid cryogen effectively to about -185 °C and maintains the temperature until observations start. The LN_2 is automatically replenished when the LN_2 level is insufficient. The cryogen injector is equipped with an electrically controlled opening/closing valve, allowing for the quick and precise release of liquid cryogen from a nozzle located at the bottom side of the cryogen injector. The valve can be operated either by an external trigger signal or manually. To ensure a sufficient flow rate of the released liquid cryogen and minimize the delay time between the input of trigger signal and the contact of released liquid cryogen on a sample, nitrogen gas is used to pressurize liquid cryogen (pressure applied above atmospheric pressure: 0.02 MPa). Our cryogen injector is affixed to a swing arm and can be easily and quickly moved off and back on a microscope stage. This design allows for easy sample handling and manipulation before observations.

The timing precision of cryogen injection was validated by measuring delay time between the trigger signals and temperature changes, which was recorded by a 25 μm diameter K-type thermocouple placed on a coverslip in an on-stage freezing chamber (Fig. 2A). The standard deviation of the measured delay times confirmed the timing precision of cryogen injection. Additionally, when configuring the timing of sample freezing in experiments, we took into consideration this delay time. Note that this measurement utilized the same apparatus employed for temperature measurement (Fig. S3 and S28).

A nanosecond pulsed laser system with a center wavelength of 355 nm (Spectra-Physics, EONE-355-1YHY-W) was used as the light source for the optical stimulus. The timing of UV light output was controlled by directly applying trigger signal to the laser system from the function generator (Fig. 2A). UV light and excitation light from a mercury lamp for widefield fluorescence imaging were combined with a dichroic mirror and subsequently introduced into the microscope. UV light was focused on the sample with a 0.7 NA dry objective lens (Nikon, CFI S Plan Fluor ELWD 60XC) with approximately 50% transmittance at a wavelength of 355 nm. Fluorescence signals were collected with the same objective lens, and fluorescence images were recorded with an sCMOS camera (Hamamatsu Photonics, ORCA-Flash4.0 V3). The excitation and detection wavelength bands for the widefield fluorescence observations were 464-499 nm (Semrock, FF01-482/35-25) and 516-556 nm (Semrock, FF01-536/40-25), respectively.

Signal-to-noise ratio (SNR) evaluation

In Fig. 3A, we evaluated the improvement in the SNR under cryogenic conditions using the data shown in Fig. 1B. As described in the figure caption, to generate a fluorescence image with an exposure time equivalent to 10 s, we integrated 1000 fluorescence images with an exposure time of 10 ms under cryogenic conditions, and then calculated the SNR under the assumption that noise follows a Poisson distribution. Although we evaluated the SNR of the fluorescence image with an exposure time equivalent to 10 s here, it is feasible to extend the measurement time under cryogenic conditions for the further improvement of SNR.

Hyperspectral fluorescence imaging of HeLa cells expressing YC3.60

HeLa cells expressing YC3.60 were observed using a hyperspectral slit-scanning fluorescence microscope

with line illumination[11] (Fig. S19). During hyperspectral imaging, HeLa cells were stimulated with histamine and then frozen rapidly, capturing the intracellular free Ca^{2+} concentration change occurring upon histamine stimulation. Trehalose was not added to the buffer solution in this experiment.

HeLa cells were immersed in HBSS buffer before histamine stimulation, and then HBSS buffer containing 10 μM histamine was added for histamine stimulation. After gentle pipetting of the HBSS buffer containing histamine and a subsequent waiting time of 30 s, the HBSS buffer was removed, and the sample was frozen rapidly. The excitation intensity was 6.8 μW on the sample plane and the exposure time per line was set to 10 ms to achieve an image acquisition time of 4 s in this experiment, which is relatively slow, but still allows us to observe the oscillation of fluorescence intensities induced by histamine stimulation.

Prior to image reconstruction from the measured fluorescence spectra of YC3.60, we measured the baseline offset value (bias level) of the EMCCD camera with its shutter closed, and then subtracted it from the measured fluorescence spectra. Fluorescence images were reconstructed using the averaged fluorescence signals at the wavelength bands where ECFP and Venus had their fluorescence peaks. The spectral bands chosen for the fluorescence images of ECFP and Venus were 475-492 nm and 517-534 nm, respectively. To reduce the remaining background signals, the average intensity values of regions without HeLa cells were obtained in each reconstructed intensity image, and the estimated background signals were then subtracted from each reconstructed intensity image. Then, YFP/CFP ratio images were generated using MetaMorph software (Molecular Devices).

Multimodal imaging using fluorescence 3D-SIM and slit-scanning Raman microscopy

HeLa cells labeled with SPY-555-actin (Spirochrome, SC202) were observed with the developed microscope (Fig. S24). The sample was mounted on a custom-made cryostage with a cooling/heating function (Linkam Scientific) (Ref. 51 of the main text) and rapidly frozen with liquid propane (-185 °C). To prevent frost condensation during rapid freezing and subsequent observation at cryogenic temperatures, N_2 gas was introduced into the space between the sample and objective lens prior to rapid freezing. Following rapid freezing, the sample temperature was controlled with the cryostage, and the liquid propane (boiling point: -42 °C) was removed from the sample by vaporization under the temperature setting of the cryostage at -40 °C. This is because it was not possible to detect Raman signals from HeLa cells under the existence of propane, which provides significantly stronger Raman signals than HeLa cells. Although we used a mixture of liquid isopentane and propane for rapid freezing in the other experiments, propane alone was used in this experiment due to the requirement of vaporization after freezing (the boiling point of isopentane at 28 °C would be too high to vaporize). Trehalose was not added to the buffer solution in this experiment. It is important to note that, our previous study demonstrated that, after rapid freezing, raising the sample temperature to -40 °C does not lead to the formation of large ice crystals, allowing the sample to remain observable without noticeable damages in optical microscopy observations like those presented in this paper and Ref. 51 of the main text. Maintaining sample temperature below -40 °C is crucial to prevent significant ice recrystallization which could result in the formation of large ice crystals (Ref. 15 of the main text).

In this multimodal imaging, fluorescence 3D-SIM and spontaneous Raman images were acquired sequentially. For fluorescence 3D-SIM imaging, the sample was illuminated with a laser light at a wavelength of 561 nm (intensity: 35.8 kW cm^{-2}). Fifteen raw images were obtained at three different illumination angles and five different phases per angle. The total image acquisition time was 750 ms. After 3D-SIM imaging, the same 561-nm excitation light was illuminated on the sample for 675 s to photobleach the fluorescent probe and reduce the strong and broad fluorescence backgrounds in Raman imaging. Raman signals of the HeLa cells were detected under line illumination with laser light at a wavelength of 532 nm. The excitation intensity was 300 kW cm^{-2} and the exposure time was 10 s $line^{-1}$. Raman images were reconstructed after removing the noise and bias background components by applying singular value decomposition (SVD) to the data obtained[12]. For the Raman images shown in Fig. 4, a stripe correction method[12, 13] was applied to correct unexpected horizontal stripes caused by defective pixels in the spectrophotometer.

Supporting figures

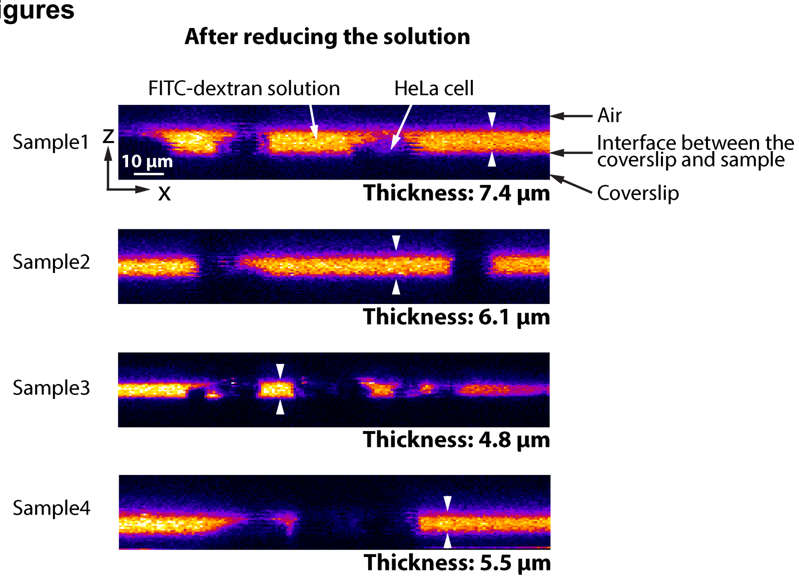

Fig. S1. Residual solution amounts in cellular samples after the reduction of HBSS buffer solution by aspiration using a pipette (representative results). The images were acquired 40-sec after pipetting, which is a typical timing ranges for freezing samples in our experiments. To visualize the HBSS buffer solution surrounding the cells, FITC-dextran 2000 (TdB lab, FD2000, 100 µg mL^{-1}) was added to the buffer solution. XZ images were acquired using a laser-scanning confocal fluorescence microscope with a 1.2 NA water immersion objective lens (Nikon, CFI Plan Apo VC 60X WI) under 473-nm excitation. At this time point, the measured residual liquid thickness was 6.7 ± 2.5 µm (n = 9, including cell thickness).

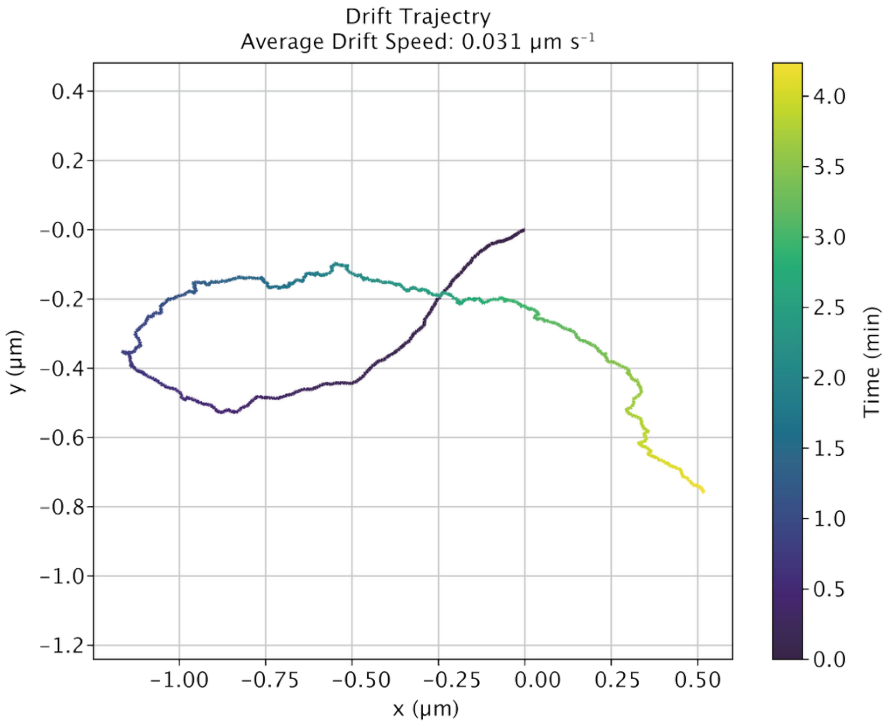

Fig. S2. 2D trajectories of fluorescent beads immobilized on a coverslip under cryo-conditions. Despite being immobilized, the beads exhibited positional shifts, most likely due to sample drift by temperature increases during observation. Although the drift was observed, the average drift velocity (31 nm s^{-1}) was sufficiently small relative to the spatial resolution and image acquisition time of fluorescence microscopes used in this study. The 2D trajectory was calculated by the cross-correlation of the phase components derived in Fourier transformation of the image series. In this experiment, imaging was performed using a 0.7 NA dry objective lens (Nikon, CFI S Plan Fluor ELWD 60XC), and the fluorescence signal was detected using an sCMOS camera (Hamamatsu Photonics, ORCA Flash4.0 V3) mounted on the side port of the microscope. The excitation wavelength was 488 nm, and the emission was detected in the 516-556 nm wavelength range (Semrock, FF01-536/40-25).

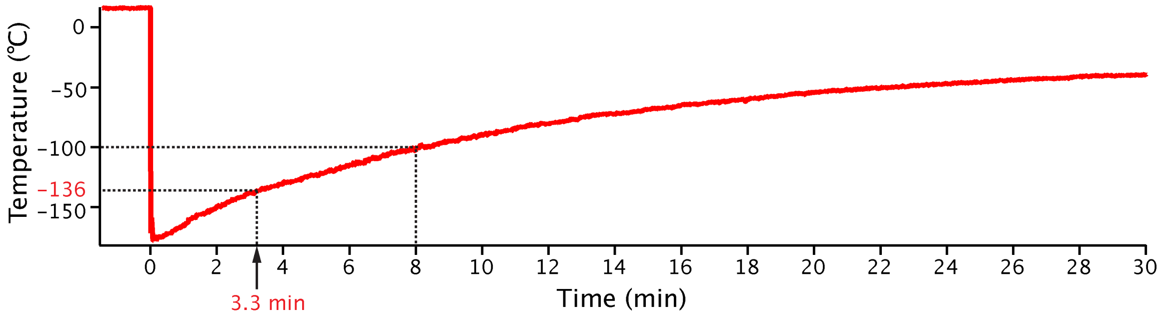

Fig. S3. Long-term temperature change after introducing liquid cryogen (a mixture of liquid isopentane and propane, temperature: about -185 °C) into the on-stage freezing chamber. The temperature was measured every 0.5 s. The result indicates that the temperature was maintained below -136 °C for about 3.3 min and -100 °C for about 8 min after freezing. For this temperature measurement, a 25 μm diameter type-K thermocouple (ANBE SMT Co., KFT-25-100-100) was used. The thermocouple was placed on a coverslip in an on-stage freezing chamber and was immersed with pure water. The thermocouple was connected to a thermocouple amplifier electrical circuit (Analog Devices, AD8495) with a theoretical cutoff frequency of 8 kHz and the signal were recorded with our homemade software.

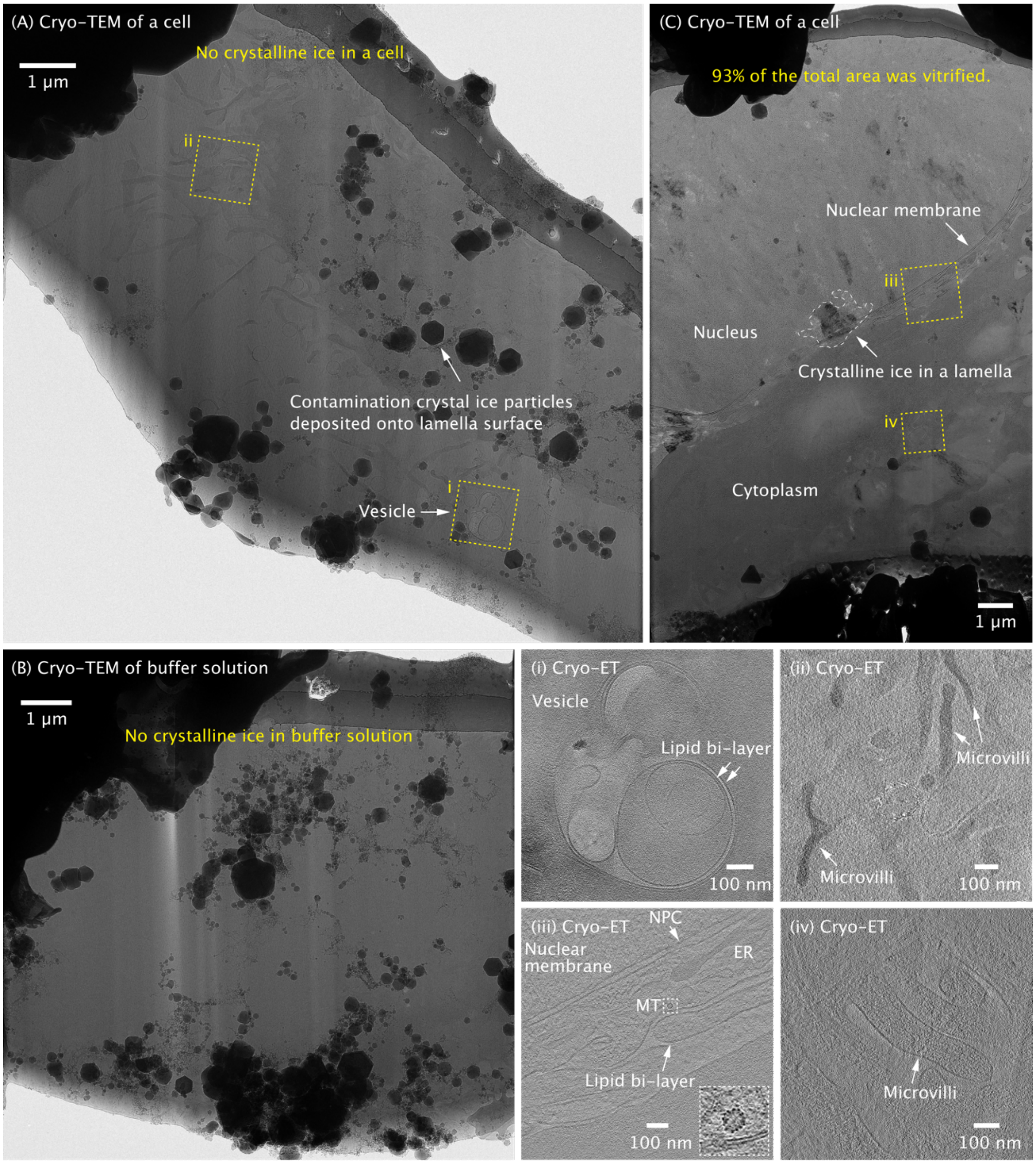

Fig. S4. Cryo-TEM and cryo-ET images of HeLa cells on a TEM grid frozen by pouring isopentane and propane mixture. No crystalline ice was observed in the two lamellae in the top left (A) and bottom left (B) panels, which contained a cell and buffer solution. The lamella shown in the top right panel (C) was mostly vitrified, with approximately 93% of the total area in vitreous state. To calculate the ratio of vitrified area in this lamella, regions of crystalline ice were manually segmented, and one of them is indicated by a white dotted line. The four panels in the bottom right show cryo-ET tomographic slices of cellular structures corresponding to rectangular areas highlighted in the cryo-TEM images, demonstrating the cellular structures were well preserved in vitreous ice. The bottom-right panel in (iii) shows an enlarged cross-sectional view of a microtubule. MT: microtubule; NPC: nuclear pore complex; ER: endoplasmic reticulum.

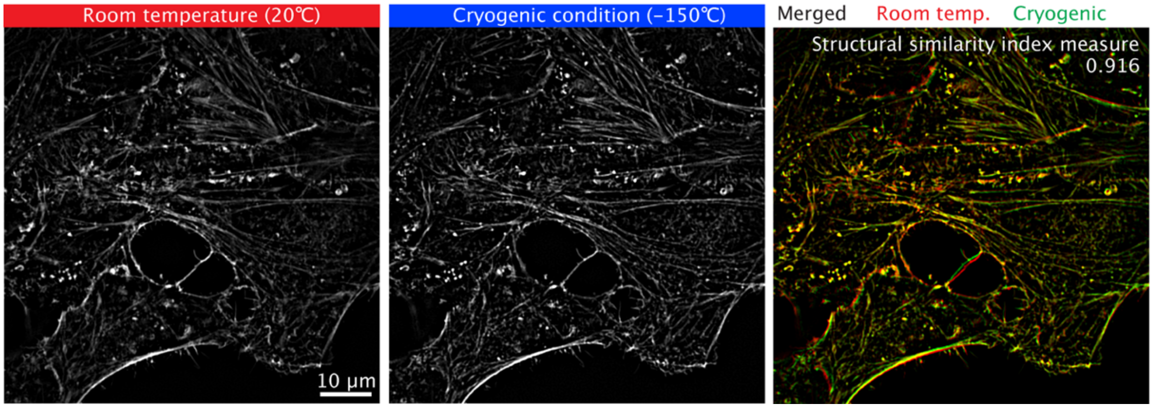

Fig. S5. Evaluation of morphological alternation at room temperature (20 °C) and under the cryogenic condition (-150 °C). COS-7 cells stained with ATTO488-phalloidin (ATTO-TEC) were fixed by paraformaldehyde (PFA) in prior to rapid freezing to avoid the morphological alteration by cellular motion and were observed by structured illumination microscopy (SIM). The cells were mounted in an on-stage freezing chamber and immersed in the PBS buffer solution at room temperature after PFA fixation and recorded (left panel). Subsequently, the PBS buffer solution was removed using a pipette, and a liquid cryogen (mixture of isopentane and propane at -185 °C) was introduced into the on-stage freezing chamber. A second set of SIM data was then acquired under the cryogenic condition at -150 °C, without any optical adjustments or modifications to the correction collar of the objective lens (middle panel). The structural similarity index measure[14] was employed to assess the morphological consistency between the images acquired under the two temperature conditions, yielding a value of 0.916. The result indicates a high degree of structural preservation under cryogenic conditions.

Imaging was performed using a 2D-SIM system equipped with a 0.7 NA dry objective lens (Nikon, CFI S Plan Fluor ELWD 60XC). Excitation was provided by a 488 nm laser source (Coherent, Genesis MX488-1000), and fluorescence signals were detected using an sCMOS camera (Hamamatsu Photonics, ORCA Flash4.0 V3) mounted on the side port of the microscope body. The optical setup of the 2D-SIM system is the same as that used in 3D-SIM, as shown in Fig. S10, except for the illumination mask.

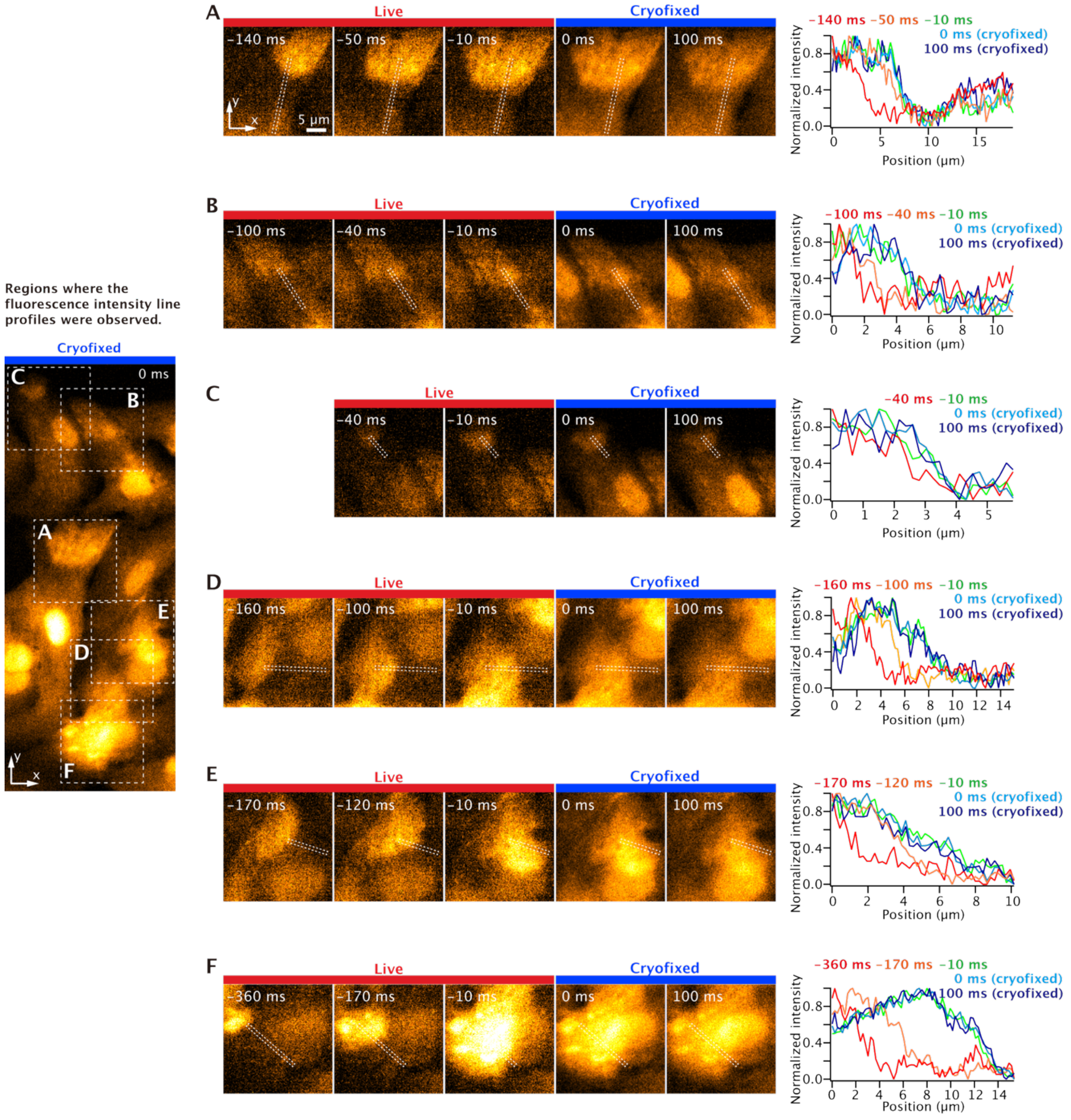

Fig. S6. Fluorescence intensity line profiles at six different positions in the fluorescence images of the neonatal rat cardiomyocytes. The fluorescence intensity profiles were obtained from the regions indicated by white dotted rectangular boxes in the images A-F. The fluorescence intensities were averaged along the short side of the white dotted rectangular box. The result confirms that rapid freezing halted the calcium ion wave propagation nearly instantly in the observed area (Video S6). In this experiment, no trehalose was added to a buffer solution.

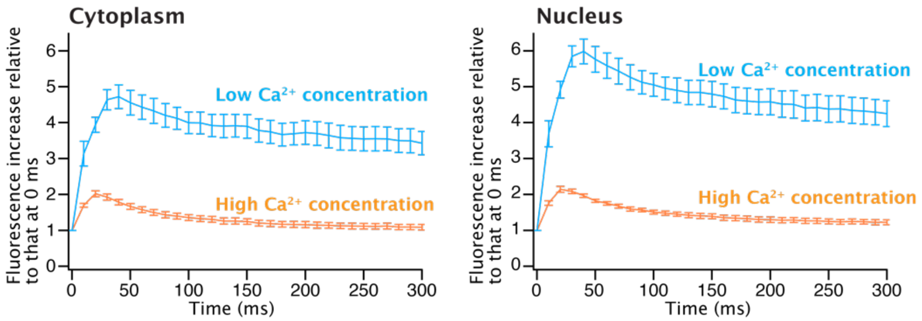

Fig. S7. Fluorescence intensities of Fluo-4 in cytoplasm and nucleus following rapid freezing. The fluorescence signals were averages at the cytoplasm and nucleus regions and normalized by the intensities at 0 ms, which were measured just before rapid freezing, while the values after 0 ms show fluorescence intensities under cryogenic conditions. The average fluorescence intensities and standard errors (shown with error bars) for low and high Ca^{2+} concentrations were calculated using the fluorescence images of 13 and 10 neonatal rat cardiomyocytes cells, respectively. Note that the data were normalized to a starting intensity of 1 for each case to allow direct comparison of relative fluorescence increases. Because the initial fluorescence intensities were lower in the low Ca^{2+} concentration regions, normalization based on these smaller values resulted in relative larger error bars compared to the high Ca^{2+} group.

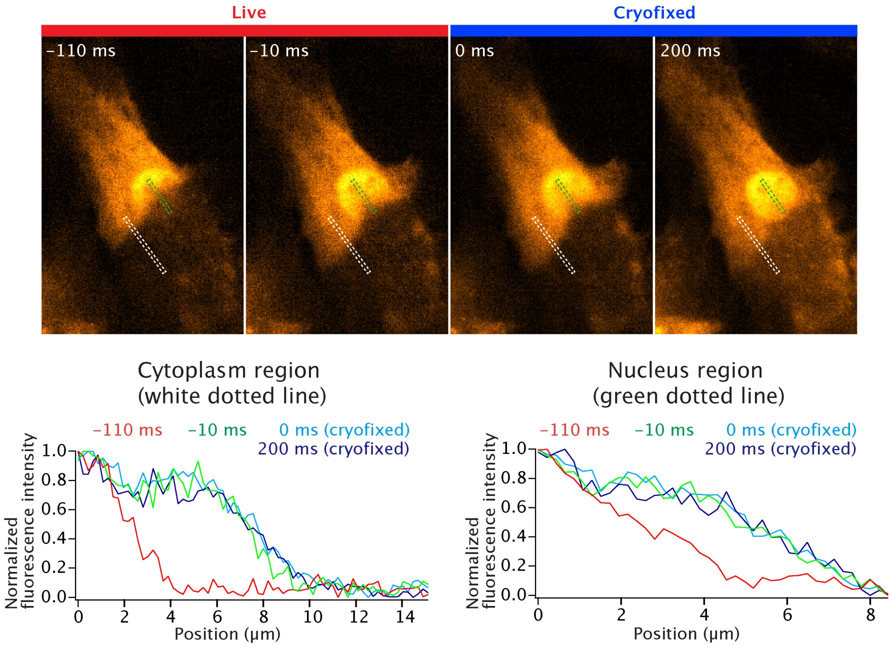

Fig. S8. Fluorescence intensity line profiles corresponding to the lines in the nucleus and cytoplasm region, indicated in white and green, in the fluorescence images of neonatal rat cardiomyocytes, which are presented in Fig. 1B. In the plot of fluorescence intensity line profiles, the contrasts in the cytoplasm and nucleus regions were independently normalized, using their respective cytoplasm and nucleus signals used as baselines. These results confirm that the propagation of Ca^{2+} wave in both the cytoplasm and nucleus regions was immobilized at the frame of 0 ms by cryofixation.

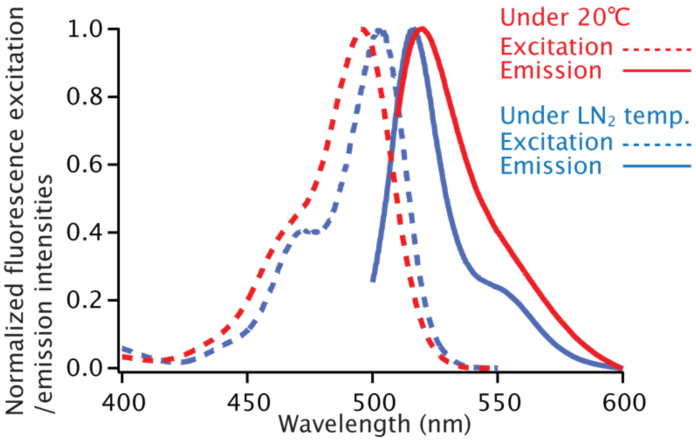

Fig. S9. Excitation and fluorescence spectra of Fluo-4 in HEPES buffer solution. The excitation and fluorescence spectra were measured with a spectrofluorometer (Hitachi, F-7000). The measurement of those spectra under LN_2 temperature was performed with an optional unit for measurements at low temperature (Hitachi, 5J0-0112/4J1-0104).

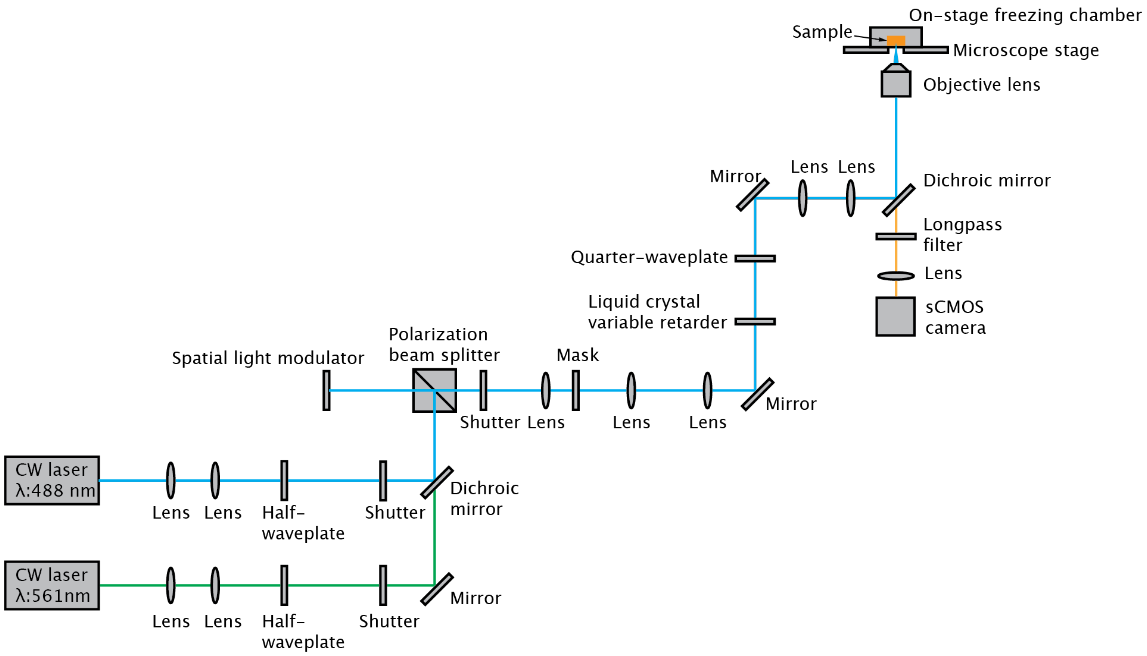

Fig. S10. Dual color 3D structured illumination microscope (3D-SIM). The configuration of the optical setup for this microscope is similar to that previously reported in Ref. 12 of the main text. The excitation wavelengths are 488 nm (Coherent, Genesis MX488-1000) and 561 nm (Spectra Physics, Excelsior 561). In this setup, a fluorescence image is acquired separately at each excitation wavelength. The polarization angle of the excitation beam is adjusted with the half-waveplate so that the excitation beam is reflected by the polarization beam splitter to a reflective spatial light modulator (SLM) (Forth dimension displays, SXGA-3DM) with 1280×1024 pixels. Periodic patterns for structured illumination displayed on the SLM produce diffracted beams. The diffracted excitation beams pass through the polarization beam splitter, and then only the 0th and $\pm 1^{st}$ order diffracted excitation beams pass through the mask placed at the focus position of the lens. The phases of the diffracted excitation beams were adjusted to maximize the contrast of the structured illumination formed on the sample plane by using a liquid crystal variable retarder (Thorlabs, LCC1413-A) and a quarter waveplate. For this SIM, we used a Nikon Ti2-E inverted microscope equipped with the automatic axial-drift compensation system (Nikon perfect focus system). The diffracted excitation beams were introduced into the microscope body and reflected to the objective by a dichroic mirror (For 488 nm excitation: Di03-R488-t3-25x36 (Semrock), 561 nm excitation: Di03-R561-t3-25x26 (Semrock)). The excitation beams were focused on the pupil plane of the objective lens. A 0.7 NA dry objective lens (Nikon, CFI S Plan Fluor ELWD 60XC) was used for Fig. 1E and 1F. Fluorescence signals were detected with an sCMOS camera (Hamamatsu Photonics, ORCA Flash4.0 V3) mounted on the side port of the microscope body.

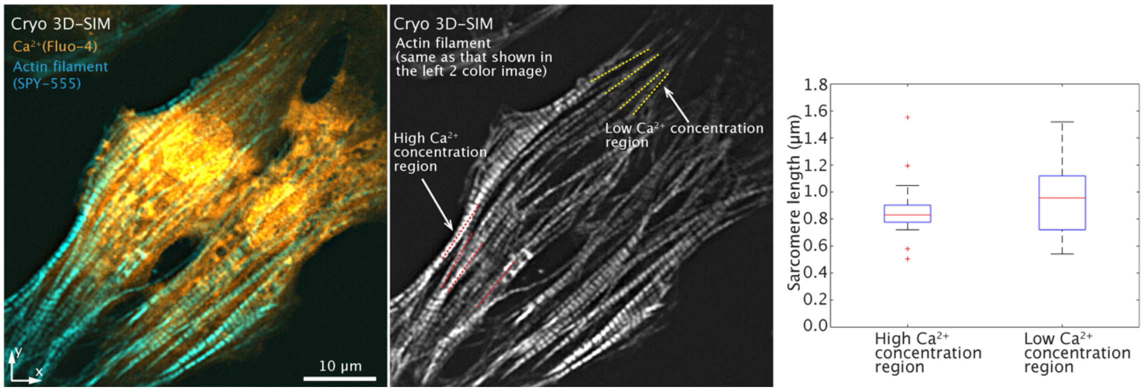

Fig. S11. Sarcomere lengths in a cryofixed neonatal rat cardiomyocyte. We examined the sarcomere lengths in the cryofixed neonatal rat cardiomyocyte of the dual color SIM data used for Fig. 1E. The sarcomere lengths were measured in 4 actin filaments in each of the low and high Ca^{2+} concentration regions, as indicated by the yellow and red dotted lines indicated in the fluorescence image of actin filaments. Our analysis showed that the sarcomere lengths were slightly shorter in the high Ca^{2+} concentration region compared to the low Ca^{2+} concentration region. The box plot shows the distribution of sarcomere lengths in high and low Ca^{2+} concentration regions and outliers are indicated by the red crosses.

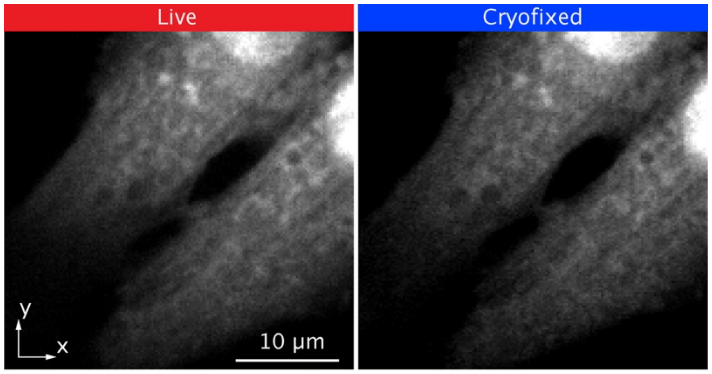

Fig. S12. Fluorescence images showing the distribution of Ca^{2+} indicator, Fluo-4, in neonatal rat cardiomyocytes before and after cryofixation. The sample was the same as that for Fig. 1E and were cryofixed during observation using a widefield fluorescence microscope equipped with a mercury lamp (Nikon, Ti2-E) at a framerate of 100 frames s^{-1}, prior to obtaining cryogenic 3D-SIM and widefield fluorescence images shown in Fig. 1E. The fluorescence image taken before cryofixation shows non-uniform fluorescence intensity distributions, including dark spots, which remained largely unchanged after cryofixation. This result confirms that these fluorescence intensity distributions, including dark spots, were already present under living conditions and were not caused by cryofixation. These distributions are considered due to the accumulation or deposition of Fluo-4 within cells (Ref. 47 in the main text), which is often observed in Fluo-4 loaded neonatal rat cardiomyocytes. Here, to improve the visibility of fluorescence intensity distributions within cells before and after cryofixation, three image frames captured just before freezing (at time points: -30, -20, -10 ms, with rapid freezing timing defined as occurring at 0 ms) and three image frames captured under cryogenic conditions were averaged, respectively. Note that, due to the beating motion, including shape changes, of the neonatal rat cardiomyocytes in this sample, it is difficult to increase the number of fluorescence image frames used for averaging while ensuring minimal motion artifacts in the resultant averaged image. As described in the main text and the Materials and Methods section, this sample was labeled with the Fluo-4 (Ex/Em: 495/518 nm), and the actin probe SPY555 (Ex/Em: 555/580 nm). To predominantly visualize Fluo-4 distribution in this experiment, the appropriate optical filters were used for the widefield fluorescence imaging; the excitation and detection wavelengths were 464-499 nm (Semrock, FF01-482/35-25) and 516-556 nm (Semrock, FF01-536/40-25), respectively. Samples were observed with a 0.7 NA dry objective lens (Nikon, CFI S Plan Fluor ELWD 60XC). Fluorescence signals were detected with an sCMOS camera (Hamamatsu Photonics, ORCA Flash4.0 V3) placed at the side port of the microscope body.

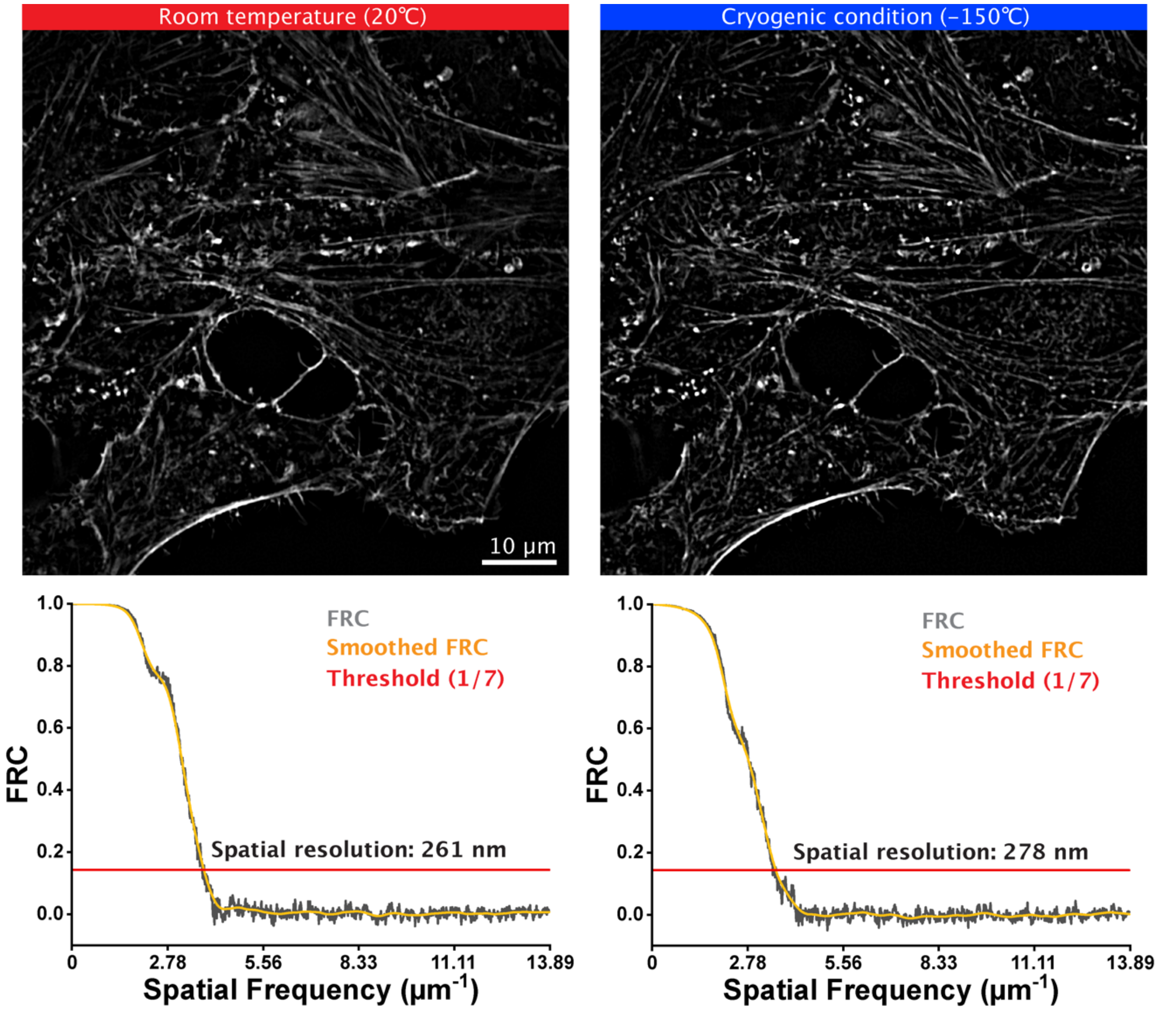

Fig. S13. Evaluation of spatial resolution before and after cryofixation. The sample and data used for this analysis are identical to those presented in Fig. S5. Practical spatial resolution of SIM was evaluated using Fourier Ring Correlation (FRC), which requires two independent samplings of the same field of view (FOV). Accordingly, two image frames were acquired per condition. FRC analysis was performed using an ImageJ plugin[15] integrated into the BioImaging and Optics Platform (BIOP). As required by the plugin documentation, the images were cropped to 2048×2048 pixels before analysis. The upper panel shows representative images acquired under each condition, while the lower panel presents the corresponding FRC plots. In the FRC graphs, the raw FRC curve, smoothed FRC curve, and the commonly used 1/7 resolution threshold are shown in gray, orange, and red, respectively. Based on the FRC analysis, the practical spatial resolutions before and after cryofixation were determined to be 261 nm and 278 nm, respectively. The difference in spatial resolution is less than the size of a single pixel in the image and is considered to be within the margin of error.

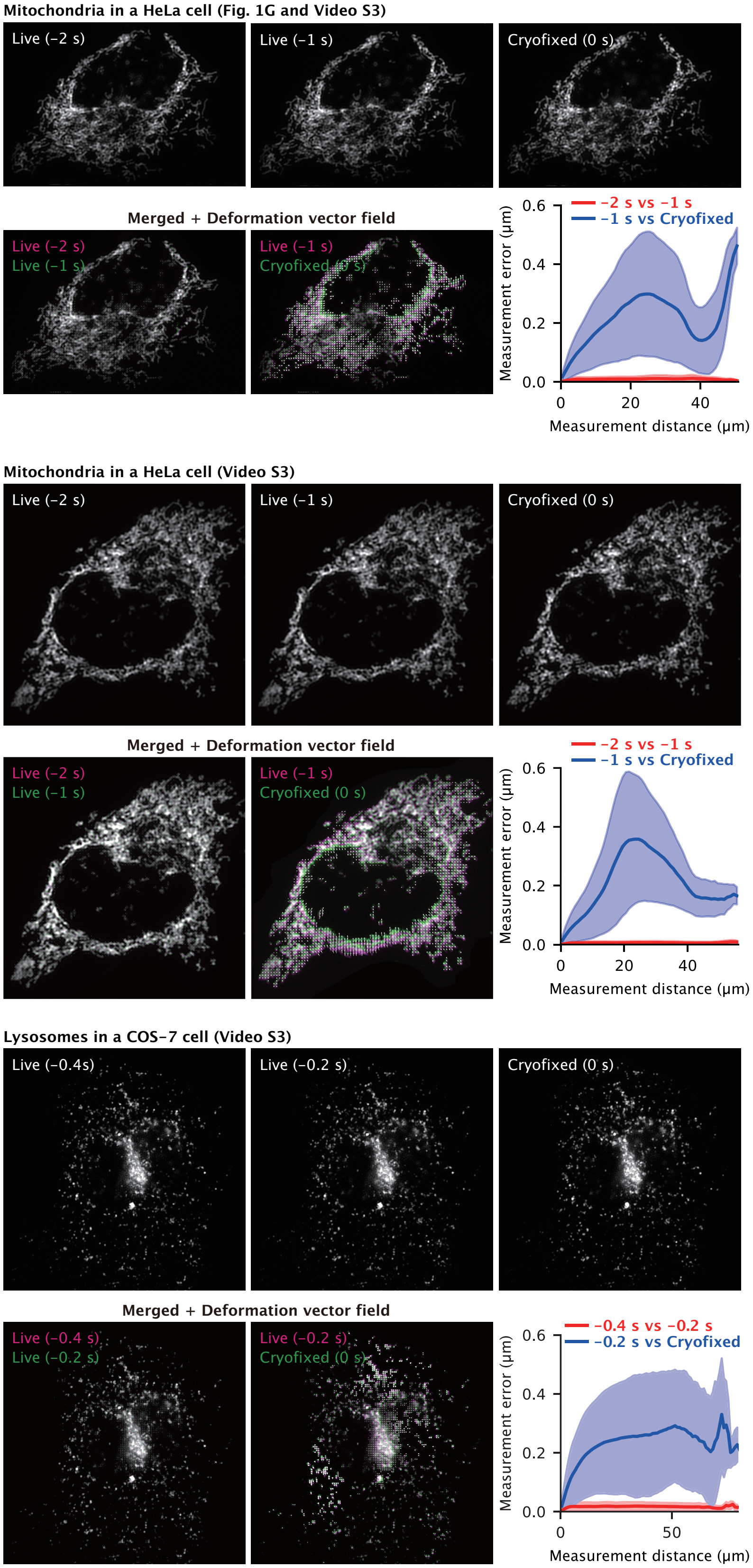

Fig. S14. Evaluation of the extent of morphological changes by cryofixation in HeLa cells expressing DsRed in mitochondria (Fig. 1G, Video S3) and COS-7 cells labeled with LysoTracker Red NDN-99 (Thermo Fisher

Scientific, L7528) (Video S3). To evaluate the extent of morphological changes caused by cryofixation, we calculated the deformation vector field and its magnitude using a B-spline-based non-rigid registration algorithm[16,17]. Prior to this calculation, Wiener deconvolution was applied to the images to sharpen the structures. Based on the calculated deformation vector field, the measurement errors in the cryofixed image were calculated by comparing various two-point length measurements within the sample before and after cryofixation (Ref. 48 of the main text). These measurement errors were then plotted as a function of the measurement length in the sample before cryofixation (blue or red line, mean; shaded area, standard deviation). The merged images and deformation analysis of the last two live frames show almost no cellular motion during image acquisition of these images, indicating that the morphological changes between the live and the cryofixed frames primarily reflect deformations caused by freezing.

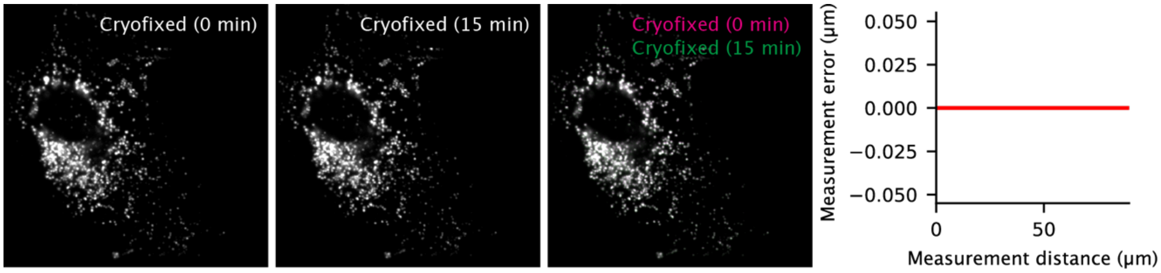

Fig. S15. Comparison of lysosomal distributions in a HeLa cell stained with LysoTracker Red NDN-99 (Thermo Fisher Scientific, L7528) immediately after freezing and again after 15 min. In the merged image, signals obtained immediately after freezing and 15 min later are shown in magenta and green, respectively; thus, regions with perfect overlap appear white, indicating no detectable change in lysosomal distribution. To quantitatively evaluate the extent of morphological changes, measurement errors were calculated using the same method described in Fig. S14, confirming no observable difference of lysosomal distribution between the two time points. The temperature at 15 min after freezing was measured to be -69 °C. This result indicates that, once a sample is rapidly frozen, large ice crystals that severely damage cellular morphology are not formed, even if the temperature subsequently increases to -69 °C, at least within the spatial resolution of a few hundred nanometers.

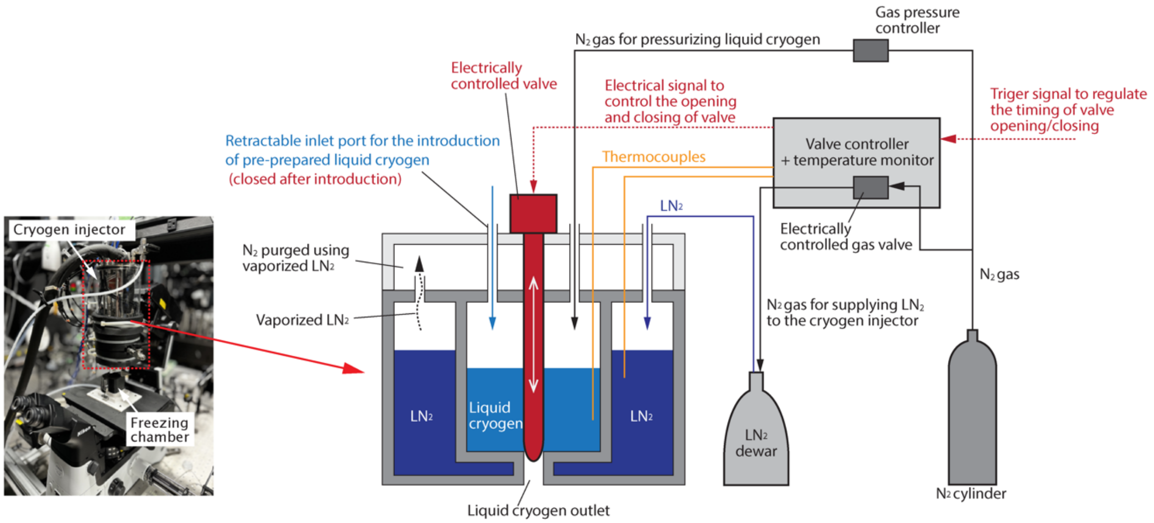

Fig. S16. Electrically controlled cryogen injector and its control system. Before the introduction of a pre-prepared mixture of liquid isopentane and propane into the cryogen injector, the injector's tank for the mixed liquid cryogen was cooled down to about -185 °C using LN_2. This temperature was maintained by regulating the volume of LN_2 within the injector. LN_2 was supplied from a LN_2 dewar, and its supply was performed by introducing N_2 gas from a N_2 cylinder to the dewar. This N_2 gas flow was regulated by a gas valve in a controller by a system that monitored the temperatures of both the LN_2 and mixed liquid cryogen tanks. Once the temperature of mixed liquid cryogen tank reached around -185 °C, the pre-prepared mixed liquid cryogen was manually introduced through the retractable inlet port at the top of cryogen injector, which was then sealed. After the temperature of mixed liquid cryogen reached -185 °C, N_2 gas was introduced from the cylinder to pressurize the mixed liquid cryogen in the injector, enabling it to be injected into the on-stage sample chamber with minimal delay at any chosen timing. The opening and closing of valve in the cryogen injector can be controlled either by an external trigger signal or manually. These temperatures were monitored using K-type thermocouples.

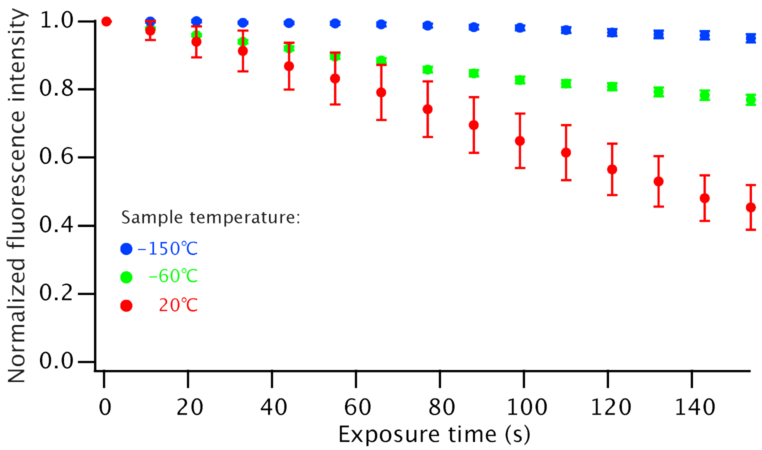

Fig. S17. Reduction of photobleaching effects of Fluo-4 under cryogenic condition. To confirm the reduction of photobleaching effects by decreasing the temperature of a sample, Fluo-4 loaded HeLa cells at different temperatures were imaged continuously for 150 s at 2 frames s^{-1} (exposure time per each image acquisition: 500 ms), and then the decrease of fluorescence signals was observed. The samples were fixed with paraformaldehyde, preventing fluorescence signal increases due to cell responses during the observation at 20 °C[18]. After paraformaldehyde fixation, samples were mounted in a customized cryostage (Linkam Scientific) with a temperature control function and then the samples were observed at 20, -60, and -150 °C. The cryostage was placed on the microscope stage of a conventional widefield inverted fluorescence microscope, and fluorescence images were recorded with a CCD camera. Fluo-4 was excited with a LED (Nikon, D-LEDI) at the excitation intensity of 2.3 W cm^{-2}. The excitation and detection wavelength bands were 465-495 nm (Nikon, excitation filter of FITC filter cube set) and 513-557 nm (Nikon, emission filter of FITC filter cube set), respectively.

The average fluorescence intensities and standard deviations were calculated from the fluorescence images of 7 HeLa cells, and the results are plotted. The error bars in the plot represents the standard deviations. From the result, we confirmed that the photobleaching effect is significantly reduced when the temperature is decreased from 20 °C to -150 °C.

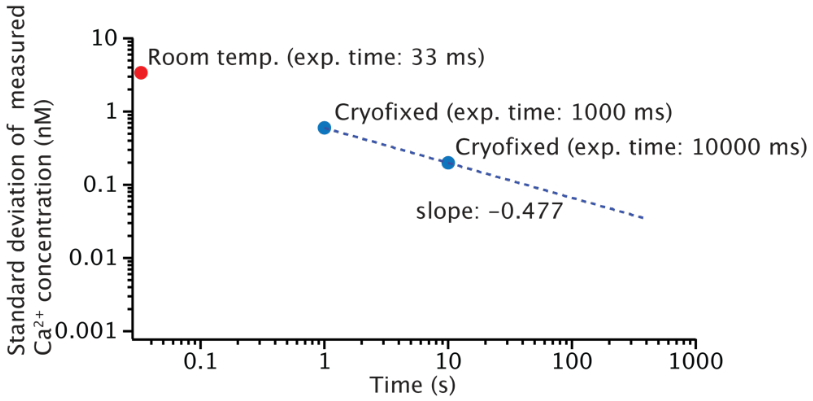

Fig. S18. Improvement of signal-to-noise ratio by increasing exposure time under cryogenic conditions. The signal-to-noise ratio for long exposure times under cryogenic conditions was evaluated by measuring fluorescence signals of Fluo-4 in free Ca^{2+} buffer solutions with a Ca^{2+} concentration of 100 nM, assuming the Ca^{2+} concentration in the cell is 100 nM. The excitation laser at 488 nm was focused into the sample solution with an 0.45 NA dry objective lens (Nikon, S Plan Fluor ELWD 20X). The excitation intensity was 55 μW μm^{-2}. The fluorescence signals were measured at 20 °C (room temperature) and -170 °C (cryofixed) with a slit confocal microscope equipped with a spectrophotometer and an EMCCD camera (Andor, iXon Ultra 888). From the measured fluorescence signals, standard deviations were calculated under the assumption that noises follow a Poisson distribution, and then the value of standard deviations were converted to Ca^{2+} concentrations (in units of nM) by performing the following calculation: standard deviation value/signal value × Ca^{2+} concentration in the sample solution (100 nM). The result indicated that the increase of the exposure time under cryogenic conditions allows us to improve the measurement accuracy of Ca^{2+} concentration. As shown in this graph, the slope of the data under cryogenic conditions was -0.477, which was obtained by fitting the data with a power function. The improvement factor of the standard deviation is nearly proportional to the square root of the increase of the exposure time. This result indicates that the fluorescence signals of cryofixed samples were measured with almost negligible photobleaching effects.

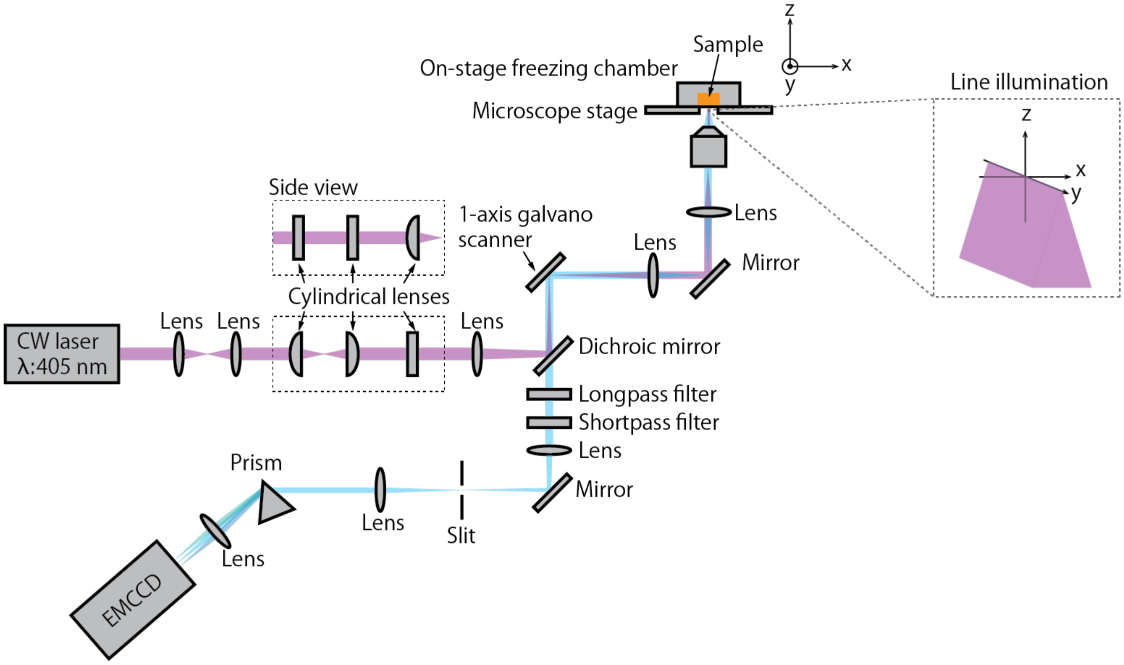

Fig. S19. Hyperspectral slit-scanning fluorescence microscope. The optical configuration of the hyperspectral slit-scanning fluorescence microscope is similar to that described in Ref. 11. The excitation wavelength was 405 nm (HÜBNER photonics, Cobolt 06-MLD). The line illumination is formed with cylindrical lenses and the x-axis scanning was performed with a single-axis galvanometer scanner. The laser beam was reflected by a dichroic mirror and introduced into a Nikon Ti2-E inverted microscope. A prism-type spectrometer was built for the spectroscopic detection of fluorescence signals (prism: Thorlabs, PS855). The spectral detection range of the spectrometer was set to span from 410-622 nm. This range was achieved by using both a longpass filter (Semrock, LP02-407RU-25) and shortpass filters (Semrock, SP01-633RU-25). Here, the shortpass filter was used to block out near-infrared light used for the autofocus system of Ti2-E inverted microscope. The excitation beam was illuminated on the sample, and fluorescence signals were collected with a 0.7 NA dry objective lens (Nikon, CFI S Plan Fluor ELWD 60XC). The fluorescence signals were recorded with an EMCCD camera (Princeton Instruments, Pro-EM:1024). Image acquisition, image reconstruction, and image processing were performed by homebuilt software.

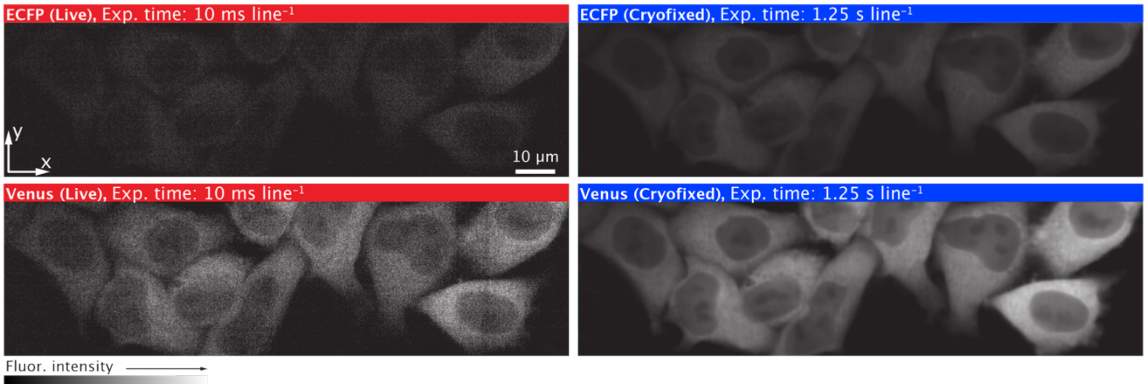

Fig. S20. Fluorescence images of HeLa cells expressing YC3.60 before and after cryofixation. The fluorescence intensity images were obtained using a hyperspectral slit-scanning fluorescence microscope shown in Fig. S19. The ratiometric fluorescence images in Fig. 3B of the main text were generated using these fluorescence intensity images. These fluorescence intensity images were reconstructed from measured fluorescence spectra and the background signals were subtracted in the manner described in Materials and Methods. Trehalose was not added to the buffer solution in this experiment.

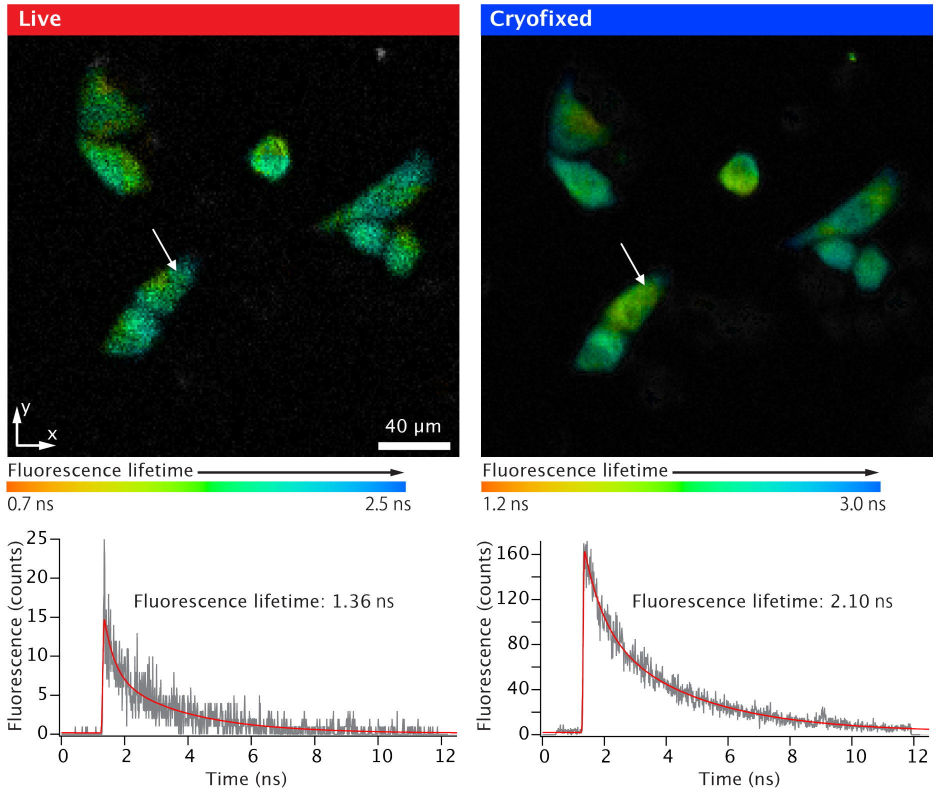

Fig. S21. Fluorescence lifetime images of HeLa cells expressing YC3.60 at room temperature and cryogenic conditions. YC3.60 was excited with a femtosecond pulse laser at a center wavelength of 405 nm and fluorescence signals of ECFP were detected in a spectral band from 466-499 nm (Semrock, FF01-483/32-25). The pixel dwell times for 20 °C and -170 °C were 100 µs and 1 ms, respectively. After cryofixation, the fluorescence lifetime became 1.54 times longer. For this measurement, a conventional confocal laser-scanning fluorescence microscope was used. The laser source was an OPO system (Spectra Physics, Inspire HF100) seeded by an 80 MHz mode-locked Ti:sapphire laser (Spectra Physics, Mai-Tai). Laser scanning was performed with a 2-axis galvanometer scanner. The excitation laser beam was focused on the sample with a 0.45 NA dry objective lens (Nikon, S Plan Fluor ELWD 20x) mounted in a Nikon Ti-E inverted microscope. Fluorescence signals were collected with the same objective lens and detected with a hybrid single-photon detector (Becker & Hickl, HPM-100). The detected fluorescence photons were counted using a time-correlated single-photon counting (TCSPC) board (Becker & Hickl, SPC-180NX). In our system, image acquisition was performed using SPCM data acquisition software (Becker & Hickl), and data analysis was performed using SPCImage NG data analysis software (Becker & Hickl). Trehalose was not added to the buffer solution in this experiment.

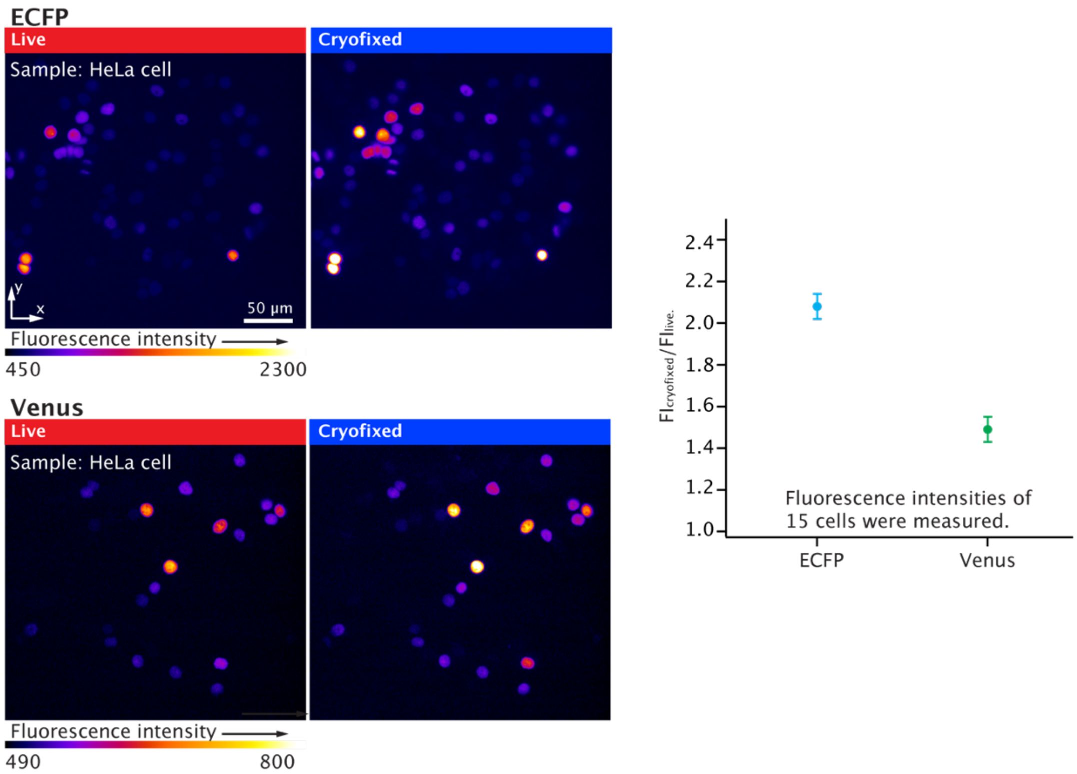

Fig. S22. Widefield fluorescence images of HeLa cells expressing ECFP and Venus in nucleus before and after cryofixation. The samples were observed with a conventional widefield fluorescence microscope equipped with a mercury lamp. From the fluorescence intensities of 15 cells in each image, we confirmed that fluorescence intensities of ECFP and Venus were enhanced 2.08 and 1.49 times under the cryogenic conditions, respectively. A 0.45 NA dry objective lens (Nikon, S Plan Fluor ELWD 20x) was used for this observation. The excitation and detection wavelength bands for ECFP were 415-455 nm (Semrock, FF02-435/40-25) and 475-495 nm (Semrock, FF01-485/20-25), respectively. The excitation and detection wavelength bands for Venus were 464-499 nm (Semrock, FF01-482/35-25) and 516-556 nm (Semrock, FF01-536/40-25), respectively. Fluorescence signals were detected with an EMCCD camera (Andor, iXon Ultra 888) with a 100 ms exposure time. Trehalose was not added to the buffer solution in this experiment. In the experiments, ECFP and Venus were expressed in the same manner as that for Fig. 1G. The construct of nuclear localization ECFP and Venus were created by replacing the Gamillus sequence of pcDNA3-Gamillus-H2B created in a previous study with the ECFP and Venus sequence, respectively[19].

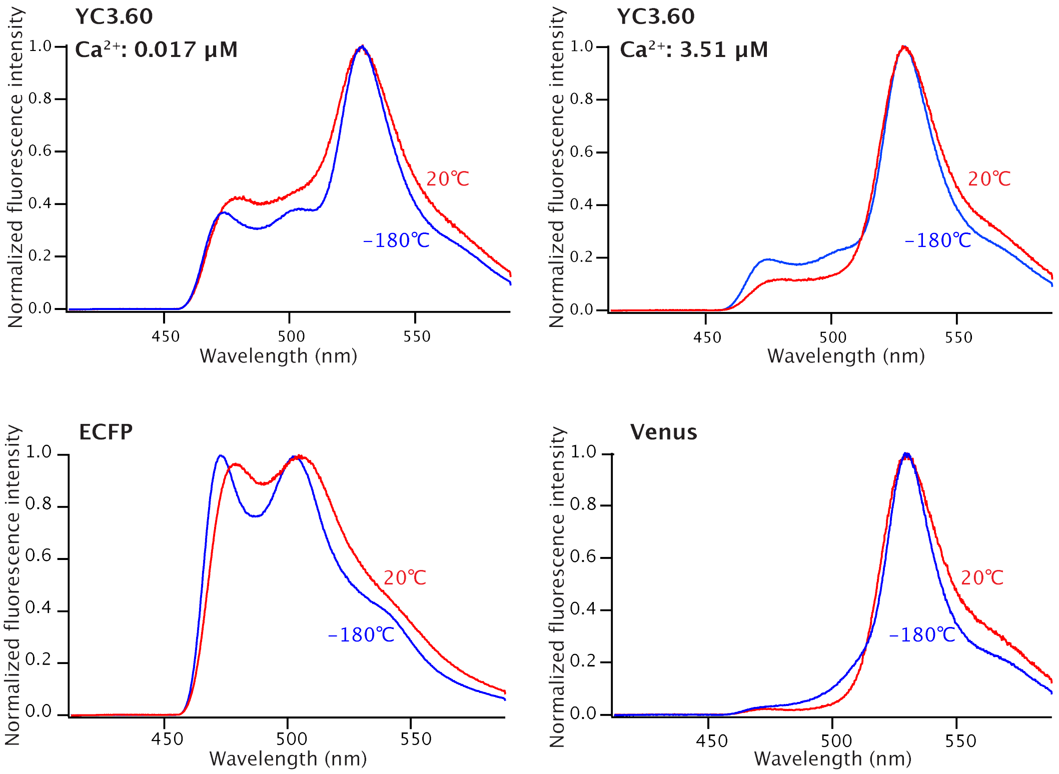

Fig. S23. Fluorescence spectra of YC3.60, ECFP, and Venus before and after cryofixation. Fluorescence spectra of YC3.60 at the Ca^{2+} concentration of 0.017 and 3.51 μM were those obtained in the experiment shown in Fig. 3D. Fluorescence spectra of ECFP and Venus were observed with a conventional widefield fluorescence microscope equipped with a light emitting diode (LED) (Thorlabs, SOLIS-405C). The change in the fluorescence spectral shape of YC3.60 between room temperature and cryogenic conditions is considered to be due to the narrowing of the spectral peaks of ECFP and Venus under cryogenic conditions. Although the shape of the fluorescence emission spectrum of YC3.60 is slightly changed by cryofixation, the fluorescence signals can be detected by using the same set of optical filters as those used at room temperature. The excitation wavelength band was 400-410 nm (Semrock, FF01-405/10-25). The fluorescence signals were detected in a wavelength region above 461 nm (Semrock, LP03-458RU-25). A 0.7 NA dry objective lens (Nikon, CFI S Plan Fluor ELWD 60XC) was used for this observation.

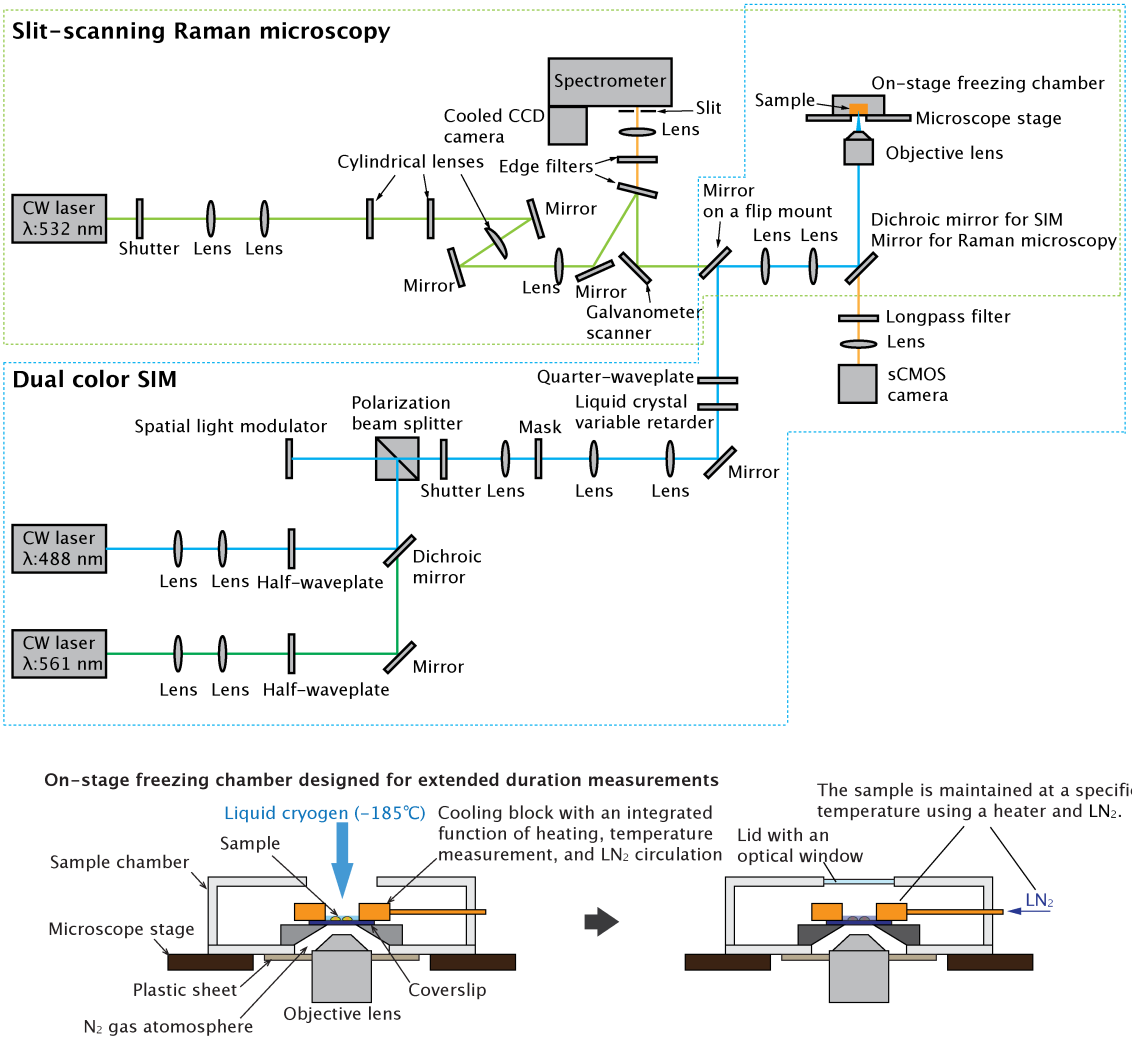

Fig. S24. Multimodal SIM/Raman microscope and an on-stage freezing chamber designed for measurement with an extended duration. To perform multimodal SIM (fluorescence) and Raman imaging[12,13,20], an optical setup for a slit-scanning Raman microscopy is introduced into the SIM (Fig. S10). The optical configuration of the Raman microscope is similar to that previously reported in Ref. 12. The excitation wavelength for Raman imaging is 532 nm (Coherent, Verdi V18). The line illumination is formed by cylindrical lenses and the x-axis scanning is performed with a single-axis galvanometer scanner. In this setup, SIM (fluorescence) and Raman images are acquired separately by switching the excitation and detection optical paths by using a mirror mounted on a flip mount and rotating a filter cube turret equipped with filter cubes of the dichroic mirror for SIM and a mirror for Raman imaging. A slit is placed at the entrance of a spectrometer (Bunkoukeiki, MK-300), and the position of the slit is conjugated to the sample plane. Spontaneous Raman signals from line illumination on a sample are recorded with a cooled CCD camera (Princeton Instruments, PIXIS:400BR). Image acquisition, image reconstruction, and image processing such as background subtraction for Raman imaging were performed by the software we have developed. A 0.95 NA dry objective

lens (Nikon, CFI Plan Apo Lambda 60XC) was used for both the line illumination of excitation light and the collection of spontaneous Raman and fluorescence signals.

For this experiment, another type of on-stage freezing chamber was used, which was designed and developed for extended duration measurement (Ref. 51 of the main text). As described in Materials and Methods section, in this freezing chamber, a sample was first rapidly frozen by the similar way as those done in other experiments in this paper, which was the introduction of liquid propane (-185 °C) into a sample from a liquid cryogen inlet port on the top of freezing chamber. Immediately after rapid freezing, the sample was maintained at cryogenic temperature by circulating LN_2 into the cooling block, and then the liquid cryogen inlet port was closed by a lid with an optical window. The sample temperature was adjusted by controlling the circulation flow of LN_2 and using the heater.

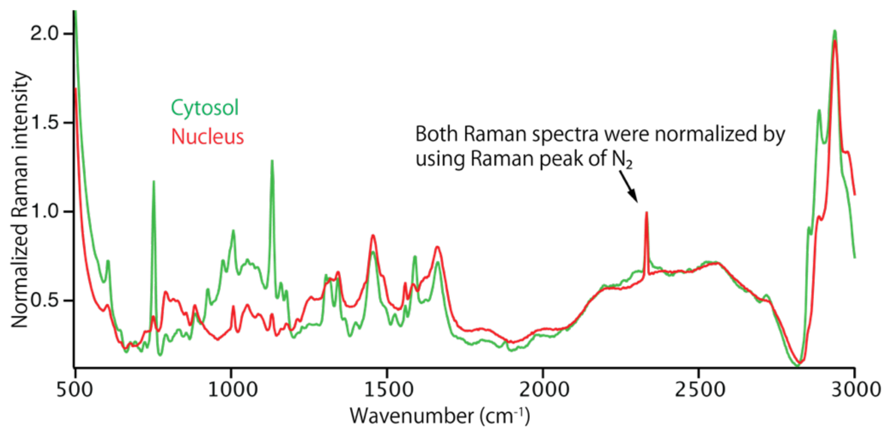

Fig. S25. Raman spectra of a HeLa cell under cryogenic conditions. These representative Raman spectra of cytosol (green) and nucleus (red) regions were obtained from a HeLa cell shown in Fig. 4 and were normalized by using the Raman peak of N_2 at 2332 cm^{-1} indicated in the figure.

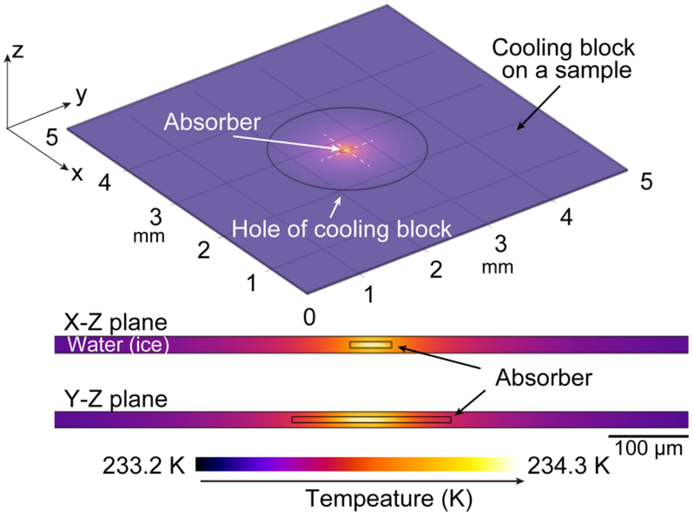

Fig. S26. Model of local heating by 10-s laser irradiation in Raman imaging of frozen cell samples. Using COMSOL Multiphysics (COMSOL Inc., version 6.2), we simulated the local temperature increase caused by laser irradiation in a sample frozen in the on-stage freezing chamber equipped with the cooling block described in Fig. S24. In the simulation, a layer of water (5 mm × 5 mm × 20 μm) was placed on a coverslip, with an absorber (50 μm × 200 μm × 7.5 μm) positioned in contact with the coverslip within the water. The absorber was assumed to be intracellular molecules (flavin adenine dinucleotide (FAD) and reduced cytochrome c) that absorb light at 532 nm. The absorption coefficients and intracellular concentrations of FAD and cytochrome c were experimentally measured and used in this simulation. For the absorber, the absorption coefficient was set to 0.022, thermal conductivity[21] to 2.40 W m^{-1} K^{-1}, heat capacity[22] to 1.80 kJ kg^{-1} K^{-1}, and density[23] to 914 kg m^{-3}. For water, the absorption coefficient[24] was 0.0004, while the thermal conductivity, heat capacity, and density were the same as those of the absorber: 2.40 W m^{-1} K^{-1}, 1.80 kJ kg^{-1} K^{-1}, and 914 kg m^{-3}, respectively. The simulation was performed under the same experimental conditions used in Fig. 4, with a sample temperature of -40 °C, 532-nm line illumination at an intensity of 300 kW cm^{-2}, and a 10-s exposure time. The result confirmed that a temperature increase was only approximately 1 °C, indicating that laser induced heating during Raman imaging is unlikely to cause thermal damage to the frozen sample.

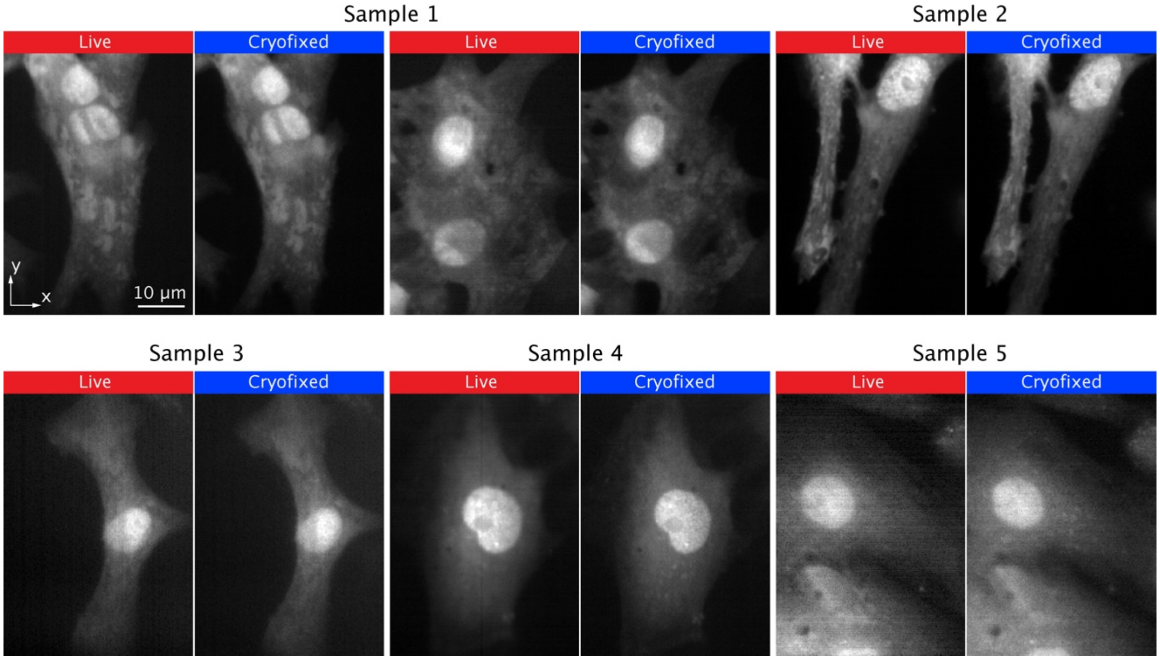

Fig. S27. Fluorescence intensity distributions and patterns in fluorescence images of neonatal rat cardiomyocytes labeled with Fluo-4 before and after cryofixation. Five different samples were observed in this experiment using a widefield fluorescence microscope equipped with a mercury lamp (Nikon, Ti2-E): Samples 1 to 4 contained 200 mM trehalose added to the buffer solution, whereas Sample 5 did not contain trehalose. Fluorescence images were captured at a framerate of 100 frames s^{-1}. In this experiment, cells that were not exhibiting dynamic motion that could alter their shape, such as beating were selected for observation. Then, to improve the visibility of fluorescence intensity distributions and patterns within cells before and after cryofixation, the recorded images at different time points were averaged. The images show that the non-uniform spatial distributions and patterns of fluorescence intensity, including dark spots, are frequently observed in Fluo-4 loaded neonatal rat cardiomyocytes under live conditions. They remain largely unchanged after cryofixation. This experiment also showed no notable difference between the samples with and without trehalose, indicating that the experiments conducted in this study can be performed without the presence of trehalose in samples. In this experiment, samples were observed with a 0.7 NA dry objective lens (Nikon, CFI S Plan Fluor ELWD 60XC). Fluorescence signals were detected with an sCMOS camera (Hamamatsu Photonics, ORCA Flash4.0 V3) mounted on the side port of the microscope body. The excitation and detection wavelengths were 464-499 nm (Semrock, FF01-482/35-25) and 516-556 nm (Semrock, FF01-536/40-25), respectively. The numbers of images used for averaging were: 1000 at room temperature and 10 after cryofixation for Sample 1 and 3; 400 at room temperature and 10 after cryofixation for Sample 2, 750 at room temperature and 10 after cryofixation for Sample 4, and 1400 at room temperature and 10 after cryofixation for Sample 5. Note that the number of images used for averaging was adjusted with the aim of achieving similar SNR level in each set of fluorescence images.

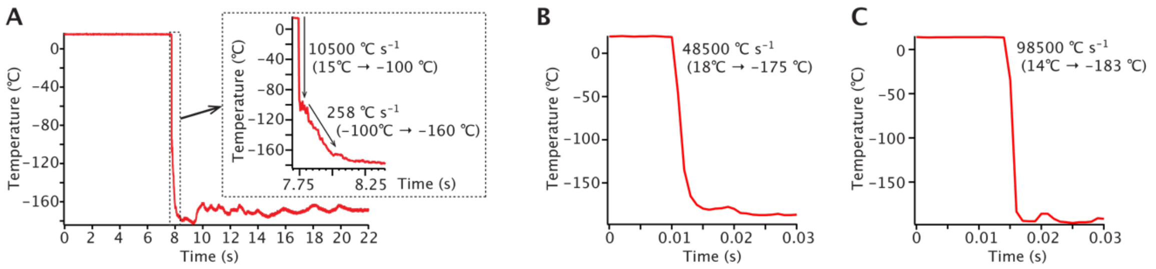

Fig. S28. (**A**) The cooling speed when approximately 40-µm-thick pure water on a coverslip was frozen by a liquid cryogen (-185 °C, a mixture of liquid propane and isopentane). (**B**) The cooling speed when the liquid cryogen was directly poured onto the thermocouple from a beaker. (**C**) The cooling speed when the cryogen was injected on the thermocouple by using the cryogen injector (Fig. S16).

Movie legends

Video S1 (separate file).

Cryofixation of Ca^{2+} wave in neonatal rat cardiomyocytes (Fig. 1B). While observing the Ca^{2+} wave in neonatal rat cardiomyocytes using a conventional inverted widefield fluorescence microscope, Ca^{2+} wave propagation was halted by cryofixation. This result demonstrates that rapid freezing can preserve not only the cell morphology and Ca^{2+} distribution, but also preserve the chemical state of the fluorescent indicator, as evidenced by the fact that the contrast of the image remains largely unaltered before and after fixation. In this experiment, we added trehalose in a buffer solution as a cryoprotectant. The concentration of trehalose was 200 mM. The image acquisition rate was 100 frames s^{-1}.

Video S2 (separate file).

(Left) Cryofixation of Ca^{2+} wave in neonatal rat cardiomyocytes without cryoprotectant. Prior to performing 3D imaging with 3D-SIM, Fluo-4 loaded neonatal rat cardiomyocytes was cryofixed under widefield fluorescence observation. As shown in the video, Ca^{2+} wave propagation was halted by cryofixation. In this experiment, cryoprotectants were not added to the buffer solution. The image acquisition rate was 100 frames s^{-1}. (Right) 3D visualization of cryofixed Ca^{2+} waves in neonatal rat cardiomyocytes (Fig. 1F). Three-dimensional Ca^{2+} distribution in the cryofixed neonatal rat cardiomyocytes, which is shown on the left in this movie, was observed using 3D-SIM. The 3D visualization was produced by using alpha rendering (Nikon, NIS-Elements).

Video S3 (separate file).

(Left) Cryofixation of mitochondria in HeLa cells (Fig. 1G). HeLa cells expressing DsRed in mitochondria were cryofixed during widefield fluorescence observation. The mitochondria motion was halted immediately after introducing the liquid cryogen for cryofixation. Although the cell shape was slightly deformed by cryofixation, it was not significant as shown in the enlarged views in Fig. 1G. In this experiment, we added trehalose in a buffer solution as a cryoprotectant. The concentration of trehalose was 200 mM. The image acquisition rate was 1 frames s^{-1}. (Right) Cryofixation of lysosomes in COS-7 cells. COS-7 cells labeled with LysoTracker Red NDN-99 (Thermo Fisher Scientific, L7528) were cryofixed under widefield fluorescence observation. Lysosome motion was halted by applying liquid cryogen. Although the slight deformation of cellular chape due to cryofixation was observed, it was not significant. In this experiment, we added trehalose in a buffer solution as a cryoprotectant. The concentration of trehalose was 200 mM. The image acquisition rate was 5 frames s^{-1}.

Video S4 (separate file).

Time-deterministic cryofixation of Ca^{2+} wave induced by uncaging Ca^{2+} from a caged Ca^{2+} compound with UV light irradiation (Fig. 2B). Neonatal rat cardiomyocytes were loaded with Fluo-4 (AAT Bioquest, 20550 or Chemical Dojin, 342-90961) and caged calcium compound (Tocris, DMNPE-4 AM-caged-calcium).

During widefield fluorescence observation, UV light was focused on a cardiomyocyte for 60 ms to uncage Ca^{2+}. Subsequently, rapidly freezing was performed at 120 ms after UV irradiation. In this experiment, we added trehalose in a buffer solution as a cryoprotectant. The concentration of trehalose was 200 mM. The image acquisition rate was 100 frames s^{-1}.

Video S5 (separate file).

(Left) Time-deterministic cryofixation of neonatal rat cardiomyocytes at the contraction phase (Fig. 2C). Neonatal rat cardiomyocytes were loaded with Fluo-4, and the increase in cytoplasmic free Ca^{2+} concentration during the contraction of the heartbeat motion, known as a Ca^{2+} transient, was observed. Cryofixation of Fluo-4 loaded neonatal rat cardiomyocytes was performed during the contraction phase. As shown in the movie, neonatal rat cardiomyocytes were frozen in time when the fluorescence intensity increased (contraction phase). In this experiment, we added trehalose in a buffer solution as a cryoprotectant. The concentration of trehalose was 200 mM. The image acquisition rate was 100 frames s^{-1}. (Right) Time-deterministic cryofixation of neonatal rat cardiomyocytes at the relaxation phase (Fig. 2D). The cryofixation of Fluo-4 loaded neonatal rat cardiomyocytes was performed during the relaxation phase. As shown in the video, neonatal rat cardiomyocytes were frozen in time when the fluorescence intensity decreased (relaxation phase). In this experiment, we added trehalose in the buffer solution as a cryoprotectant. The concentration of trehalose was 200 mM. The image acquisition rate was 100 frames s^{-1}.

Video S6 (separate file).

Cryofixation of Ca^{2+} wave in neonatal rat cardiomyocytes (Fig. S6). This video presents another demonstration of the instantaneous immobilization of Ca^{2+} wave propagations in cells by cryofixation. The experimental conditions were identical to those used for Fig. 1B, but no trehalose was added to a buffer solution in this experiment. The image acquisition rate was 100 frames s^{-1}.

References

1. K. P. Ryan and D. H. Purse, Plunge-cooling of tissue blocks: determinants of cooling rates, *J. Micros.* **140**, 47-54 (1985).
2. T. Nakagami, H. Tanaka, P. Dai, Shien-Fong Lin, Takuji Tanabe, Hiroki Mani, Katsuji Fujiwara, Hiroaki Matsubara, Tetsuro Takamatsu, Generation of reentrant arrhythmias by dominant-negative inhibition of connexin43 in rat cultured myocyte monolayers, *Cardiovasc. Res.*, **79**, 70-79 (2008).
3. T. Matsushita, M. Oyamada, H. Kurata, S. Masuda, A. Takahashi, T. Emmoto, I. Shiraishi, Y. Wada, T. Oka and T. Takamatsu, Formation of Cell Junctions Between Grafted and Host Cardiomyocytes at the Border Zone of Rat Myocardial Infarction, *Circulation* **100**, II-262–Ii-268 (1999).
4. J. W. Eastman, E. J. Rosa, The fluorescence of adenine. The effects of solvent and temperature on the quantum yield, *Photochem. Photobiol*. **7**, 189-201 (1968).
5. D. R. Haynes, A. Tokmakoff, S. M. George, Temperature-dependent absolute fluorescence quantum yield of C60 multilayers, *Chem. Phys. Lett.* **214**, 50-56 (1993).
6. B. S. Reddy and B. N. Chatterji, An FFT-based technique for translation, rotation, and scale-invariant image registration, *IEEE Trans. Image Process.* **5**, 1266-1271 (1996).
7. D. Schneidereit, H. Vass, B. Reischl, R. J. Allen, and O. Friedrich, Calcium Sensitive Fluorescent Dyes Fluo-4 and Fura Red under Pressure: Behaviour of Fluorescence and Buffer Properties under Hydrostatic Pressures up to 200 MPa. *PLoS ONE* **11**, e0164509 (2016).
8. S. B. Tikunova, J. A. Rall, and J. P. Davis, Effect of Hydrophobic Residue Substitutions with Glutamine on Ca^{2+} Binding and Exchange with the N-Domain of Troponin C, *Biochem.* **41**, 6697-6705 (2002).
9. K. Wicker, O. Mandula, G. Best, R. Fiolka, and R. Heintzmann, Phase optimisation for structured illumination microscopy, *Opt. Express* **21**, 2032-2049 (2013).
10. R. Heintzmann, V. Sarafis, P. Munroe, J. Nailon, Q. S. Hanley, T. M. Jovin, Resolution enhancement by subtraction of confocal signals taken at different pinhole sizes, *Micron* **34**, 293–300 (2003).
11. T. Kubo, K. Temma, N. I. Smith, K. Lu, T. Matsuda, T. Nagai, K. Fujita, Hyperspectral two-photon excitation microscopy using visible wavelength. *Opt. Lett.* **46**, 37–40 (2021).
12. A. F. Palonpon, J. Ando, H. Yamakoshi, K. Dodo, M. Sodeoka, S. Kawata, K. Fujita, Raman and SERS microscopy for molecular imaging of live cells. *Nat. Protoc.* **8**, 677–692 (2013).
13. K. Mochizuki, Y. Kumamoto, S. Maeda, M. Tanuma, A. Kasai, M. Takemura, Y. Harada, H. Hashimoto, H. Tanaka, N. I. Smith, K. Fujita, High-throughput line-illumination Raman microscopy with multislit detection. *Biomed. Opt. Express* **14**, 1015-1026 (2023).
14. Z. Wang, A. C. Bovik, H. R. Sheikh, and E. P. Simoncelli, Image quality assessment: from error visibility to structural similarity, *IEEE T. Image. Process.* **13**, 600-612 (2004).
15. R. P. J. Nieuwenhuizen, K. A. Lidke, M. Bates, D. L. Puig, D. Grünwald, S. Stallinga, and B. Rieger, Measuring image resolution in optical microscopy, *Nat. Methods*. **10**, 557-562 (2013).
16. K. Ntatsis, N. Dekker, V. Valk, T. Birdsong, D. Zukić, S. Klein, M Staring, M McCormick, itk-elastix: Medical image registration in Python, *Proceedings of the 22nd Python in Science Conference*, 101 – 105 (2023).
17. D. P. Shamonin, E.E. Bron, B.P.F. Lelieveldt, M. Smits, S. Klein and M. Staring, Fast parallel image registration on CPU and GPU for diagnostic classification of Alzheimer's disease, *Front. Neuroinform.* **7**, 1-15 (2014).
18. R. L. Fork, Laser stimulation of nerve cells in aplysia. *Science* **171**, 907-908 (1971).
19. H. Shinoda, Y. Ma, R. Nakashima, K. Sakurai, T. Matsuda, T. Nagai, Acid-tolerant monomeric GFP from Olindias Formosa. *Cell Chem. Biol.*, **25**, 330-338.e7 (2018).

20. K. S. Lee, Z. Landry, F. C. Pereira, M. Wagner, D. Berry, W. E. Huang, G. T. Taylor, J. Kneipp, J. Popp, M. Zhang, J.-X. Cheng, R. Stocker, Raman microspectroscopy for microbiology. *Nat. Rev. Methods Primers* **1**, 80 (2021).

21. O. Haida, T. Matsuo, H. Suga, S. Seki, Calorimetric study of the glassy state X. Enthalpy relaxation at the glass-transition temperature of hexagonal ice, *J. Chem. Thermodynamics* **6**, 815-825 (1974).

22. G. A. Slack, Thermal conductivity of ice, *Phys. Rev. B.* **22**, 3065-3071 (1980).

23. K.-H. Liu, Y. Zhang, J.-J. Lee, C.-C. Chen, Y.-Q. Yeh, S.-H. Chen, C.-Y. Mou, Density and anomalous thermal expansion of deeply cooled water condined in mesoporous silica investigated by synchrotron X-ray diffraction, *J. Chem. Phys.* **139**, 064502 (2013).

24. R. M. Pope, E. S. Fry, Absorption spectrum (380-700 nm) of pure water. Ⅱ. Integrating cavity measurements, *Appl. Opt.* **36**, 8710-8723 (1997).
